# Cadherin-11 Is Required for Neural Crest Specification and Survival

**DOI:** 10.3389/fphys.2020.563372

**Published:** 2020-10-30

**Authors:** Subrajaa Manohar, Alberto Camacho-Magallanes, Camilo Echeverria, Crystal D. Rogers

**Affiliations:** ^1^Department of Biology, School of Math and Science, California State University Northridge, Northridge, CA, United States; ^2^Department of Anatomy, Physiology, and Cell Biology, UC Davis School of Veterinary Medicine, Davis, CA, United States

**Keywords:** Cadherin-11, neural crest, specification, apoptosis, survival, p53, caspase

## Abstract

Neural crest (NC) cells are multipotent embryonic cells that form melanocytes, craniofacial bone and cartilage, and the peripheral nervous system in vertebrates. NC cells express many cadherin proteins, which control their specification, epithelial to mesenchymal transition (EMT), migration, and mesenchymal to epithelial transition. Abnormal NC development leads to congenital defects including craniofacial clefts as well as NC-derived cancers. Here, we identify the role of the type II cadherin protein, Cadherin-11 (CDH11), in early chicken NC development. CDH11 is known to play a role in NC cell migration in amphibian embryos as well as cell survival, proliferation, and migration in cancer cells. It has also been linked to the complex neurocristopathy disorder, Elsahy-Waters Syndrome, in humans. In this study, we knocked down CDH11 translation at the onset of its expression in the NC domain during NC induction. Loss of CDH11 led to a reduction of *bonafide* NC cells in the dorsal neural tube combined with defects in cell survival and migration. Loss of CDH11 increased p53-mediated programmed-cell death, and blocking the p53 pathway rescued the NC phenotype. Our findings reveal an early requirement for CDH11 in NC development and demonstrated the complexity of the mechanisms that regulate NC development, where a single cell-cell adhesion protein simultaneous controls multiple essential cellular functions to ensure proper specification, survival, and transition to a migratory phase in the dorsal neural tube. Our findings may also increase our understanding of early cadherin-related NC developmental defects.

## Introduction

Cadherin proteins are calcium dependent cell-cell adhesion molecules, which are essential for the development and maintenance of embryonic tissues (Giger and David, 2017; Taneyhill and Schiffmacher, 2017). Cadherins are single pass transmembrane proteins that contain a calcium-binding extracellular domain as well as a cytoplasmic domain which links with three catenin family proteins (α, β, and p120) and the actin cytoskeleton (Gul et al., 2017). Classical cadherins are divided into types I and II. Type I cadherins include epithelial cadherin (E-cadherin/CDH1) and neural cadherin (N-cadherin/CDH2) among others, which have been implicated in both central nervous system (CNS) development in chick and zebrafish embryos (Dady et al., 2012; Miyamoto et al., 2015), and neural crest (NC) specification and the epithelial to mesenchymal transition (EMT) in frog, fish, and chicks (Piloto and Schilling, 2010; Rogers et al., 2013, 2018; Huang et al., 2016). Type II cadherins include Cadherin 7 (CDH7), Cadherin 11 (Osteoblast-cadherin/CDH11), and Cadherin-6B (CDH6), which have been linked to CNS patterning, NC cell delamination, EMT, and migration during embryonic development (Taneyhill et al., 2007; Liu et al., 2008; Kashef et al., 2009; Schiffmacher et al., 2014, 2016). With varied timing and onset of protein expression, the regulation and function of cadherin proteins is clearly important for normal development of the CNS, NC cells, and NC derivatives.

Here, we focus on identifying the role of Cadherin-11 (CDH11) during early avian embryogenesis. CDH11 has been identified as a Wnt signaling target and effector in developmental and disease systems (Hadeball et al., 1998; Chalpe et al., 2010; Satriyo et al., 2019). Originally defined as a mesenchymal marker with no expression in the undifferentiated neural tube in mouse embryos (Hoffmann and Balling, 1995), the transcript was subsequently reported in developing mouse neuroepithelia (Kimura et al., 1995, 1996), as well as migratory NC cells in chick and frog embryos (Vallin et al., 1998; Borchers et al., 2001; Chalpe et al., 2010). It has been identified as a major regulator of NC migration in *Xenopus* embryos (Vallin et al., 1998; Kashef et al., 2009; Abbruzzese et al., 2016; Langhe et al., 2016) and has been linked to tumor growth, cell survival, and EMT in disease models (Yoshioka et al., 2015; Piao et al., 2016; Row et al., 2016). Both reduced and increased levels of CDH11 are linked to patient survival and reduced metastasis in numerous cancers; however, its role is contrasting in different cancer cell types (Carmona et al., 2012; Lee et al., 2013). Specifically, high levels of CDH11 expression have been linked to poor prognosis in gastric cancer and triple-negative breast cancer (Chen et al., 2018; Satriyo et al., 2019), yet it maintains a pro-apoptotic tumor suppressor role in others (Marchong et al., 2010; Li et al., 2012). Although studies have linked CDH11 to NC migration its role in NC induction, specification, maintenance, or survival during premigratory stages has not been studied.

NC cells are a vertebrate-specific population of stem-like cells that form craniofacial bone, cartilage, pigment cells, and the peripheral and enteric nervous systems (Hutchins et al., 2018; Rogers and Nie, 2018). In avian embryos, NC cells begin as tightly adherent neuroepithelial cells in the dorsal neural tube. By going through an EMT, which is controlled by alterations in the expression of type I and II cadherin proteins (Taneyhill et al., 2007; Rogers et al., 2013; Scarpa et al., 2015), the NC cells detach from each other and the basal lamina and gain the ability to migrate. Abnormal NC development can cause congenital defects known as neurocristopathies, which include cleft palate, craniofacial abnormalities, albinism, and defects in the enteric and peripheral nervous systems among others (Reissmann and Ludwig, 2013; Lopez et al., 2018). Bi-allelic mutations in CDH11 have specifically been linked to Elsahy-Waters syndrome, which is a combination of abnormal craniofacial developmental morphologies including those likely induced by neurocristopathies (Harms et al., 2018). The processes of NC specification and EMT are tightly regulated at multiple levels by signaling molecules (Bhattacharya et al., 2018), epigenetic modifiers (Hu et al., 2012), transcription factors (Simoes-Costa et al., 2015), and adhesion molecules (Abbruzzese et al., 2016; Schiffmacher et al., 2016; Rogers et al., 2018) to prevent developmental defects. Previous studies showed that perturbation of factors involved in this process can directly affect the formation and migratory ability of NC cells. Studies also identified links between cadherin proteins and NC cell migration and differentiation; however, there is little known about how type I or II cadherin proteins regulate premigratory NC development. Our study focuses on the role of CDH11 during the time point it first emerges in the NC domain in the dorsal neural tube. We thoroughly define the spatiotemporal localization of CDH11 in the neural plate and neural tube as well as in the pre‐ and post-migratory NC cells in the chick embryo during early stages Hamburger Hamilton (HH) stages 4–12. Loss of CDH11 expression reduces the premigratory NC population marked by PAX7, SOX9, SNAI2, and SOX10, and increases membrane-associated CDH1, F-actin, p53 and p53-mediated apoptosis in the presumptive NC regions. Our results indicate that the upregulation of CDH11 in the dorsal neural tube prior to NC migration is necessary for NC cell specification, survival, EMT, and migration.

## Results

### CDH11 Expression Starts in the Neural Tube Prior to NC Cell Formation

CDH11 function has been extensively examined during NC migration and EMT in amphibian embryos (Hadeball et al., 1998; Vallin et al., 1998; Borchers et al., 2001; Kashef et al., 2009; McCusker et al., 2009; Koehler et al., 2013; Abbruzzese et al., 2016), but less is known about its endogenous expression and role in amniotes, which encouraged us to begin by examining the spatiotemporal expression pattern of the CDH11 protein in the chicken embryo. First, protein lysate was collected at multiple developmental stages (HH4–6, HH8–10, and HH11–12) and western blot analysis was used to define the relevant stages. To test the antibody reactivity across species, we also used lysate from tailbud stage *Ambystoma mexicanum* (axolotl) whole embryos. Two antibodies against CDH11 were tested: a previously verified monoclonal mouse antibody against recombinant intracellular peptide of human CDH11 (Chalpe et al., 2010) and a rabbit polyclonal antibody directed against human CDH11 used previously in mouse tissue (Chang et al., 2017; Supplementary Figure S1A). The mouse antibody identified three bands (potentially different isoforms or posttranslationally modified versions of CDH11) between the sizes of 110–135 kD while the rabbit antibody identified only the mid-sized band and did not recognize axolotl CDH11. Further, the mouse antibody bound to an antigen in the non-neural ectoderm (Supplementary Figures S1B–E’, white arrow). Both antibodies show that CDH11 protein is expressed by stages HH4–6 through HH11–12 in chicken (Supplementary Figures S1A–M’). It is likely that the mouse antibody recognizes three versions of CDH11 (pro-, active full-length, cleaved) previously reported in *Xenopus* based on the absence of the smallest band from the early premigratory stages (McCusker et al., 2009). We also performed whole mount IHC using both antibodies, and although their expression profiles were similar in whole mount (Supplementary Figures S1B–M), in section, the mouse antibody also bound to an epitope localized in non-neural ectoderm (Supplementary Figures S1C’–E’, arrows). Due to its specificity, we chose to use the rabbit antibody for the rest of our experiments. We also verified specificity of the antibody by performing CDH11 loss and gain of function experiments and visualizing reduction or exogenous expression of the protein in whole mount (Supplementary Figures S2A–D,H–J, S3) and transverse sections (Supplementary Figures S2E–G,K–M, S3).

**Figure 1:**
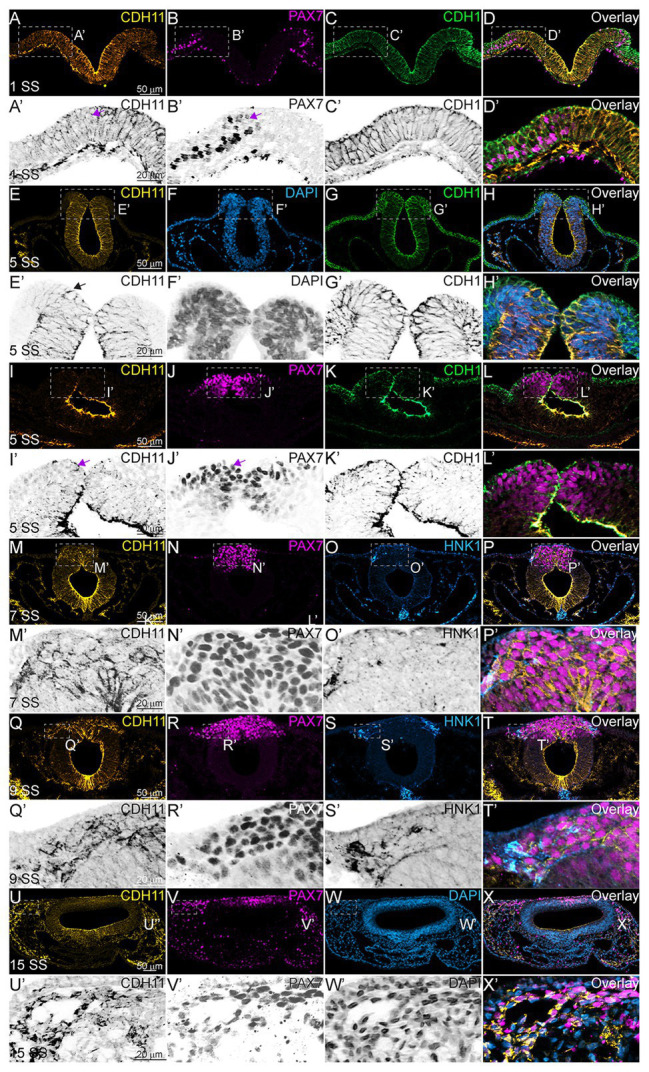
CDH11 expression in NC cells starts during specification stages. **(A–X’)** Immunohistochemistry (IHC) using antibodies against CDH11 (yellow), PAX7 (pink) to mark NC cells and neural plate border, CDH1 (green) to mark cell-cell junctions and epithelial tissues, and HNK1 (blue) to mark migratory NC cells as well as parts of the endoderm, mesoderm, and notochord, or stained with DAPI (blue) to mark nuclei. At **(A–D’)** HH7[1 somite stage (SS)], when NC are induced at the neural plate border, CDH11 is expressed in the neural plate/tube, but is only expressed in a subset of border cells with PAX7. Examples of cells positive for both PAX7 and CDH11 are marked with pink arrows. **(E-H’, I-L’)** At HH8 (5 SS), CDH11 co-localizes with CDH1 in the developing neural tube. Black arrow marks cell in premigratory NC region positive for CDH11. **(M–P’)** At HH9 (7 SS) is strongly upregulated in the premigratory NC cells marked by PAX7 and early migrating NC cells marked by HNK1. **(Q-T’)** At late HH9 (9 SS) expression is maintained in the NC cells undergoing EMT and migrating out of the neural tube. **(U–X’)** At HH11 (15 SS), CDH11 expression is weaker in the neural tube and is maintained in the migratory NC cells marked by PAX7. Dashed boxes indicate zoom regions depicted by grayscale images. Scale bars as indicated in first row.

To characterize the spatiotemporal localization of CDH11 in early avian development, we performed IHC using anti-CDH11 in conjunction with previously characterized markers of NC cells (PAX7 and HNK1; Del Barrio and Nieto, 2004; Basch et al., 2006). The results identify CDH11 expression in the neural plate of 1 somite stage (SS) embryos at HH7 (Figures 1A–D,A’–D’, Supplementary Figures S1F–M’), but is expressed at much lower levels in the neural plate border, which expresses PAX7 and CDH1 as NC cells are being induced (Figures 1B–D,B’–D’). As the neural tube begins to fuse at 5 SS (HH8). CDH11 expression is maintained throughout the neural tube, co-localizing with dorsal cells expressing CDH1 (Figures 1E–H,E’–H’). CDH11 expression starts to emerge in a subset of PAX7-positive cells in the dorsal neural tube (Figures 1I–L,I’–L’, Supplementary Figures S1H–M). At 7 SS (HH9), as NC cells begin to delaminate and undergo EMT, CDH11 is upregulated in the most proximal PAX7-positive cells (Figures 1M–P,M’–P’). As HNK1 expression begins in the early migrating NC cells, the leading cells co-express CDH11 (Figures 1O,P,O’,P’). In 9 SS (late HH9) embryos, as NC cells begin to migrate away from the midline, and all migratory cells are positive for both CDH11 and PAX7 (Figures 1Q-T,Q’–T’), while the most lateral cells express HNK1 (Figures 1S,T). Focusing on CDH11-postive cells at 7 SS (Figure 1M’) and 9 SS (Figure 1Q’) shows that CDH11 appears membrane-localized as NC cells collectively migrate out of the neural tube. At 15 SS (HH11), CDH11 remains in the neural tube and the migratory NC cells, co-localizing with PAX7 (Figures 1U–X,U’–X’); however, the cellular localization in later migratory NC appears more punctate (Figures 1Q’–T’). Our results support previous studies in frog by demonstrating the endogenous expression of CDH11 in migrating chick NC cells (McCusker et al., 2009; Abbruzzese et al., 2016; Mathavan et al., 2017). These data confirm that CDH11 is expressed during NC cell EMT and migration, but introduce novel expression in epithelial premigratory NC cells suggesting that CDH11 may play an earlier role in NC development.

### CDH11 Is Necessary for NC Cell Population Maintenance During Specification

**Figure 2:**
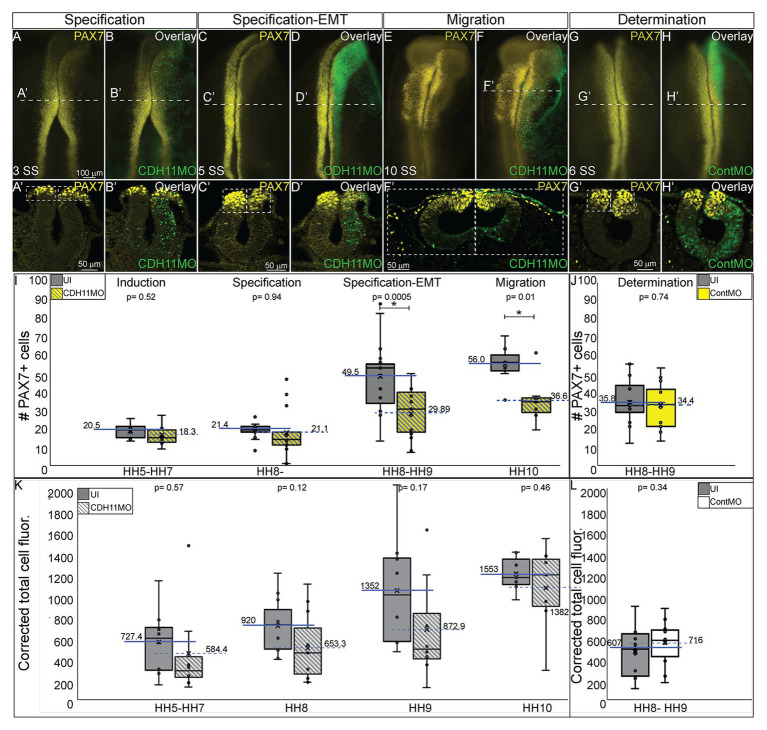
CDH11 is required for NC cell population specification. Embryos were injected at HH4 and collected at multiple stages to analyze NC progenitor marker, PAX7. **(A-F’)** IHC for PAX7 on embryos injected with CDH11MO and collected between 3 SS and 10 SS. **(G–H’)** IHC for Pax7 in embryo injected with ContMO and collected at 6 SS. **(I,J)** Actual PAX7+ cell counts of uninjected and morpholino-injected sides. **(K)** Corrected total cell fluorescence of PAX7 expression in section images injected with either CDH11MO or ContMO. **(A)** Whole mount IHC for PAX7 in HH8‐ (3 SS) embryo with **(B)** overlay with CDH11MO (green). **(A’,B’)** Transverse section of **(A,B)** with PAX7-positive NC cells circled. Mean number of cells at HH8‐ is 21.43 on uninjected and 21.14 on CDH11MO-injected side, *p* = 0.94, *n* = 14. **(C)** Whole mount IHC for PAX7 in HH8 embryo with **(C)** overlay with CDH11MO (green). **(C’,D’)** Transverse section of **(C,D)** with PAX7-positive NC cells boxed. Mean number of cells at HH8 is 49.53 on uninjected and 29.89 on CDH11MO-injected side, *p* = 0.0005, *n* = 19. **(E)** Whole mount IHC for PAX7 in HH10 embryo with **(F)** overlay with CDH11MO (green). **(F’)** Transverse section of (F) with PAX7-positive NC cells. Mean number of cells at HH10 is 56.00 on uninjected and 36.57 on CDH11MO-injected side, *p* = 0.01, *n* = 7. **(G)** Whole mount IHC for PAX7 in HH8 embryo with **(H)** overlay with ContMO (green). **(G’,H’)** Transverse sections of **(G,H)** with PAX7-positive NC cells circled. Mean number of cells on uninjected side is 35.8 and on ContMO-injected side is 34.4, *p* = 0.74, *n* = 14. Dashed boxes were drawn around NC cell population from uninjected side and are mirrored on injected sides to demonstrate changes in the NC cell population density. At 5 SS the NC cell population is less dense in the CDH11MO-side compared to uninjected when compared to 3 SS and ContMO-injected embryos. **(K,L)** Fluorescence intensity calculated using NIH ImageJ (see methods) from HH5 (*n* = 8, *p* = 0.57), HH8 (*N* = 11, *p* = 0.11), HH9 (*n* = 8, *p* = 0.17), HH10 (*n* = 7, *p* = 0.46), and ContMO HH8–9 (*n* = 12, *p* = 0.34). Scale bars are as marked (100 μm for whole mount and 50 μm for sections). Anterior to top in all whole mount images, dorsal to top in all sections. Loss of CDH11 reduces the PAX7-positive NC cell population after induction (**I**, HH5-3SS) and at a point between specification and determination (5 SS).

Next, to understand the stage at which CDH11 is necessary for NC cell development, and to determine whether CDH11 was required for induction, specification, or maintenance of the NC population, in addition to its role in migration, a time-course experiment was performed. We used a translation-blocking CDH11MO, which effectively reduced CDH11 fluorescence intensity on the injected side of the embryo compared to the uninjected side by approximately 50% (Supplementary Figures S2A–G, S3). CDH11 expression was inhibited at gastrula stage (HH4), prior to the expression of the neural plate border marker, PAX7. After injection, embryos were collected at stages HH5 and HH7 and IHC for PAX7 was performed. We next counted the number of PAX7-positive cells and measured fluorescence intensity on the morpholino-injected side compared to the uninjected side. As expected, at NC induction and early specification stages prior to the onset of CDH11 expression upregulation (HH5‐ HH8-), the number of PAX7-positive NC progenitors in the neural plate border was unchanged (Figures 2A–B’,I, *n* = 14, *p* = 0.94). In contrast, at 5 SS (HH8), when CDH11 expression emerges in the dorsal neural tube, the number of PAX7-positive NC cells was reduced by 40% (Figures 2C–D’, *n* = 19, *p* = 0.0005). At 10 SS (HH10), there continued to be 35% less PAX7-positive cells on the CDH11MO-injected side, suggesting that the NC cells did not recover prior to migration (Figures 2E–F’,I, *n* = 7, *p* = 0.01). Embryos injected with a non-specific control morpholino (ContMO), did not exhibit significant differences in the number of PAX7-positive cells between the injected and uninjected sides (Figures 2G–H’, *n* = 14, *p* = 0.74). We additionally assessed the changes in fluorescence intensity at each stage after CDH11 knockdown and although the fluorescence was reduced in the injected vs. uninjected sides, due to variability in the NC population responses to CDH11MO, the changes in fluorescence were not significant (Figure 2K, *p* > 0.05). Taken together, loss of CDH11 significantly reduced PAX7+ NC cells after induction during the stages at which NC specifier genes and proteins are normally upregulated suggesting a role for CDH11 specifically in NC specification or maintenance in preparation for EMT, prior to its role in NC migration.

### Loss of CDH11 Reduces Expression of NC Specifiers SNAI2, SOX9, and SOX10

**Figure 3:**
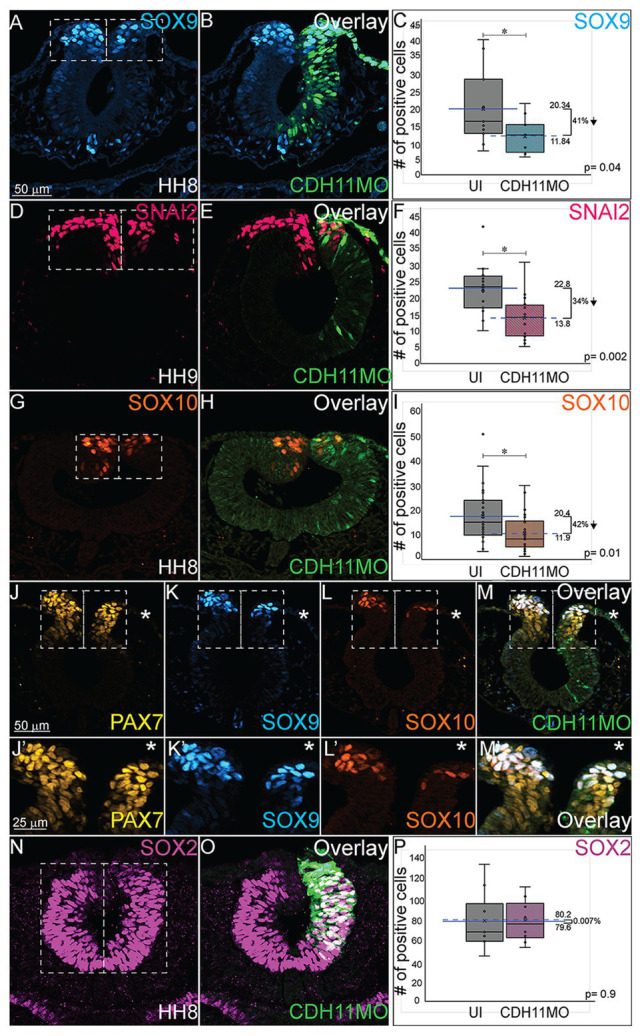
Loss of CDH11 reduces definitive NC cells. To determine if loss of CDH11 affected neural progenitors and definitive NC cells in addition to NC progenitors, embryos were injected with CDH11MO or ContMO and electroporated, and IHC was performed for definitive NC cells (SOX9, SNAI2, SOX10) and neural progenitors (SOX2). **(A)** IHC for SOX9 in transverse section from HH8 embryo with **(B)** overlay with CDH11MO (green). **(C)** Graph showing difference between uninjected and CDH11MO-injected sides. Mean number of SOX9+ cells is 20.34 on uninjected and 11.85 on CDH11MO-injected side, *p* = 0.04, *n* = 11. **(D)** IHC for SNAI2 in transverse section from HH9 embryo with **(E)** overlay with CDH11MO (green). **(F)** Graph showing difference between uninjected and CDH11MO-injected sides. Mean number of SNAI2+ cells is 22.75 on uninjected and 13.75 on CDH11MO-injected side, *p* = 0.001, *n* = 16. **(G)** IHC for SOX10 in transverse section from HH8 embryo with **(H)** overlay with CDH11MO (green). **(I)** Graph showing difference between uninjected and CDH11MO-injected sides. Mean number of SOX10+ cells is 20.35 on uninjected and 11.87 on CDH11MO-injected side, *p* = 0.01, *n* = 18. **(J–M’)** Overlay from the same embryo to demonstrate reduction in NC progenitors (PAX7) and definitive NC cells (SOX9, SOX10) after CDH11 MO-injection (*n* = 8/9 embryos with reduced cells). **(N)** IHC for SOX2 in transverse section from HH8 embryo with **(O)** overlay with CDH11MO (green). **(P)** Graph showing difference between uninjected and CDH11MO-injected sides. Mean number of SOX2+ cells is 80.20 on uninjected and 79.50 on CDH11MO-injected side, *p* = 0.90, *n* = 5. All graphs show mean (indicated on graph) and median (line within graph). Scale bar for (A,B,D,E,G,H,N,O) indicated in (A) and scale bar for (J-M’) indicated in (J). Reducing CDH11 significantly reduces the entire population of premigratory NC cells without affecting the SOX2-positive neural tube progenitors.

As reported by multiple groups, in the NC gene regulatory network (GRN) the factors in the neural plate border (i.e., PAX7, PAX3) drive the expression of bonafide NC markers (SNAI2, SOX9, SOX10, etc.) as neurulation proceeds (Basch et al., 2006; Plouhinec et al., 2014; Williams et al., 2019). These factors are then responsible for altering the expression of specific cadherin proteins and allowing for NC cell EMT and migration (Taneyhill et al., 2007; Huang et al., 2016; Taneyhill and Schiffmacher, 2017). To determine if loss of CDH11 universally reduced the NC cell population by reducing definitive NC cells, embryos were unilaterally injected with CDH11MO, electroporated at HH4, and IHC was used to detect bonafide NC cell markers (SOX9, SNAI2, SOX10) at HH8–9 (5 SS to 7 SS). Loss of CDH11 significantly reduced SOX9-postive NC cells by 41% (Figures 3A–C, *n* = 11, *p* = 0.04). SNAI2-positive cells were reduced by 34% (Figures 3D–F, *n* = 16, *p* = 0.001) and SOX10-positive NC cells were reduced by 41.7% (Figures 3G–I, *n* = 19, *p* = 0.01). We next knocked down CDH11 and performed IHC for PAX7, SOX9, and SOX10 in the same embryos to confirm that the loss of CDH11 was affecting both NC cell progenitors and the premigratory *bonafide* NC population. We identified that both cell populations were reduced in the absence of CDH11 (Figures 3J–M’, *n* = 8/9). Finally, we wanted to determine if the CDH11-knockdown phenotype was NC-specific or if loss of CDH11 affected all ectodermal derivatives. Therefore, we analyzed the expression of SOX2, a neural tube progenitor marker, which was unaffected in the CDH11MO-innjected side compared to the UI-side (Figures 3N–P, *n* = 5, *p* = 0.9).

The loss of CDH11 thus reduces the amount of progenitors and definitive NC cells prior to NC cell migration. However, each NC specifier protein drives specific programs with regards to NC cell development. SNAI2 inhibits *Cdh6B* and *Cdh1* expression to drive cell migration (Taneyhill et al., 2007; Tien et al., 2015) and it is also linked to the inhibition of apoptotic activity in NC cells (Tribulo et al., 2004), while the SOXE proteins, SOX9 and SOX10 are linked with the progression of NC cell migration (Cheung and Briscoe, 2003). To determine the mechanisms downstream of CDH11 knockdown that led to a reduction in the NC population, we next analyzed the impact on cell death and proliferation.

### CDH11 Is Required for NC Cell Survival

**Figure 4:**
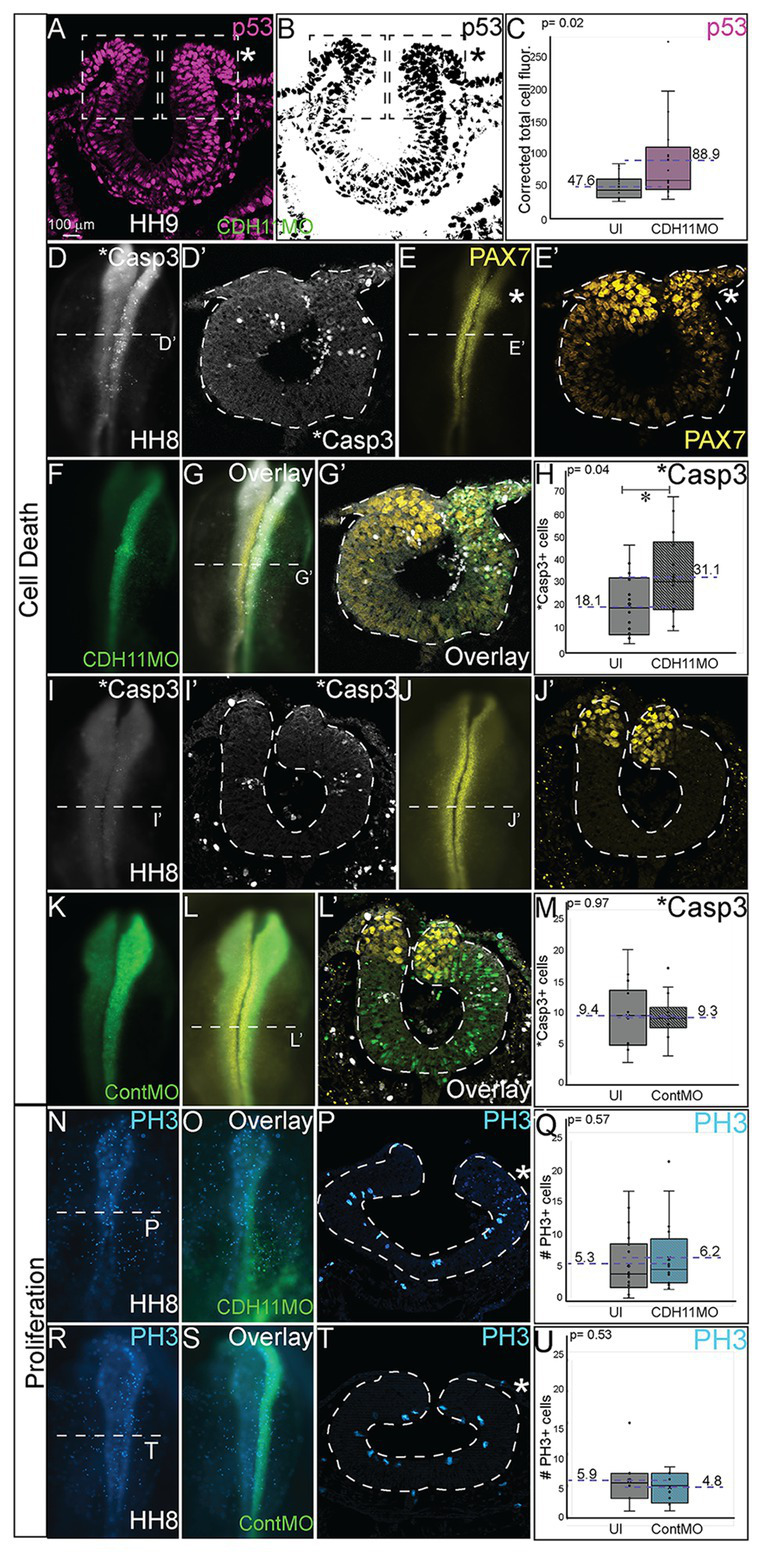
Loss of CDH11 increases cell death. To determine the cause of reduced NC cell population after CDH11 knockdown, IHC was performed for **(A-C)** p53, **(D,D’,G,G’,I,I’,L,L’)** activated caspase 3 (*Casp3) to mark apoptotic cells, **(E,E’,G,G’,J,J’,L,L’)** PAX7, or **(N–U)** phosphorylated histone H3 (PH3) to mark mitotic cells. **(A,B)** IHC for p53 in transverse section. **(C)** Graph showing difference between fluorescence intensity on uninjected and CDH11MO-injected sides (*n* = 17, *p* = 0.02). **(D)** IHC for *Casp3 or **(E)** PAX7 in whole mount or for **(D’)** *Casp3 or **(E’)** PAX7 in transverse section, **(F)** CDH11MO in whole mount and **(G**,**G’)** are overlays. **(H)** Graph showing increased number of *Casp3-positive cells on CDH11MO side. Mean number *Casp3+ apoptotic cells is 17.90 on uninjected and 31.86 on CDH11MO-injected side, *n* = 14, *p* = 0.04. **(I)** IHC for *Casp3 or **(J)** PAX7 in whole mount or for **(I’)** *Casp3 or **(J’)** PAX7 in transverse section, **(K)** ContMO in whole mount and **(L**,**L’)** are overlays. **(M)** Graph showing difference between uninjected and ContMO-injected sides. Mean number of *Casp3+ apoptotic bodies is 9.36 on uninjected and 9.36 on ContMO-injected side, *p* = 0.97, *n* = 14. **(N)** IHC for PH3 in whole mount and **(O)** overlay with CDH11MO. **(P)** Transverse section from HH8 embryo injected with CDH11MO on right side (green). **(Q)** Graph showing difference between uninjected and CDH11MO-injected sides. Mean number of PH3+ cells is 6.20 on uninjected and 5.20 on CDH11MO-injected side, *p* = 0.50, *n* = 20. **(R)** IHC for PH3 in whole mount embryo injected with **(S)** ContMO. **(T)** Transverse section. **(U)** Graph showing difference between uninjected and ContMO-injected sides. Mean number of PH3+ cells is 5.88 on uninjected and 4.75 on ContMO-injected side, *p* = 0.54, *n* = 8. Loss of CDH11 increases cell death on injected side. All graphs show mean (indicated on graph) and median (line within graph). Scale bar whole mount images indicated in (A) and for sections in (C). Asterisk indicates injected side in sections.

The reduction in NC cells after CDH11 knockdown could be caused by two cellular responses. To determine if this phenotype was a result of increased cell death (or reduced cell survival) or of a reduction NC cell proliferation (or reduced population growth), we unilaterally injected chicken embryos with CDH11MO or ContMO, electroporated at HH4, and performed IHC for markers of cell death and cell proliferation. To determine the requirement for CDH11 in NC cell survival, IHC was performed for tumor protein p53 (p53) as previous work in chicken NC cells demonstrated that p53 plays a role in NC cell EMT and that excess p53 reduces the premigratory NC cell population (Rinon et al., 2011). The fluorescence intensity of p53 expression was measured in the dorsal region of the neural tube, and in the absence of CDH11, p53 expression was increased in the CDH11MO-injected side of the neural tube compared to the uninjected side (Figures 4A–C, *n* = 17, *p* = 0.02, Supplementary Figures S4A–D). We next analyzed expression of the p53-mediated apoptosis effector protein, activated Caspase-3 (*Casp3) together with PAX7 (Zou et al., 1999). After CDH11 knockdown, the total number of *Casp3-positive cells was counted in the injected and uninjected sides of each embryo. Expression of *Casp3 was significantly increased by 71.5% in CDH11MO-injected sides compared with the uninjected side (Figures 4D,D’,G,H, *n* = 14, *p* = 0.04), concurrent with a reduction in PAX7-positive cells (Figures 4E,E’,G,G’). However, embryos injected with ContMO showed no significant difference in *Casp3 or PAX7 between the injected versus uninjected sides (Figures 4I-M, *n* = 14, *p* = 0.97). We also performed terminal deoxynucleotidyl transferase dUTP nick end labeling (TUNEL) analysis to further confirm the presence of apoptotic cells in the neural tube after CDH11 knockdown. We identified an increase in fluorescence intensity of the TUNEL staining in the injected side of CDH11MO-injected neural tubes compared to the uninjected side (Supplementary Figures S4E–H,J, *n* = 12, *p* = 0.02) while ContMO-injected embryos had no significant difference in TUNEL staining between the injected and uninjected sides (Supplementary Figure S4I, *n* = 5, *p* = 0.53). Loss of CDH11 is thus correlated with an increase in cell death and CDH11 may be necessary for NC cell survival.

Previous results in *Xenopus* embryos linked loss of CDH11 to increased Wnt-dependent cell cycling and suggested that loss of CDH11 was positively correlated with NC cell proliferation (Koehler et al., 2013). To determine if NC cell proliferation was altered after CDH11 knockdown at the premigratory stage, we injected and electorporated CDH11MO or ContMO unilaterally at HH4 and performed IHC for phosphorylated histone H3 (PH3), a marker of mitotic cells (Figures 4N–U). We determined that neither CDH11MO (Figures 4N–Q, *n* = 20, *p* = 0.57) nor ContMO (Figures 4R–U, *n* = 8, *p* = 0.54) significantly changed the number of PH3-positive cells suggesting that there is no consistent role for CDH11 in NC proliferation. These data demonstrate a requirement for CDH11 in NC cell survival.

### Blocking p53-Mediated Apoptosis Rescues NC Cells

**Figure 5:**
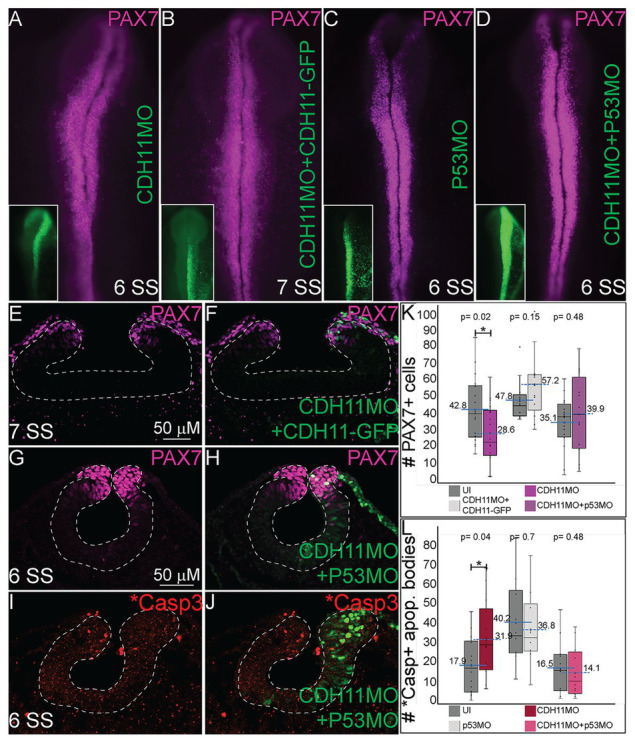
Blocking p53-mediated apoptosis rescues the NC fate. To determine if the NC and *Casp3 phenotypes resulted from p53-mediated apoptosis, embryos were injected with multiple combinations of treatments to attempt to rescue the phenotype. **(A–D)** Whole mount IHC for PAX7 in HH8‐ HH9 embryos after **(A)** CDH11MO, **(B)** CDH11MO + CDH11-GFP, **(C)** p53MO, or **(D)** CDH11MO + p53MO. Inset shows treatment injection (green). **(E)** IHC for PAX7 in transverse section from HH9 embryo with **(F)** overlay with CDH11MO + CDH11-GFP (green). **(G)** IHC for PAX7 in transverse section from HH9 embryo with **(H)** overlay with CDH11MO + p53MO (green). **(I)** IHC for *Casp3 in transverse section from HH8 embryo with **(J)** overlay with CDH11MO + p53MO (green). **(K)** Graph showing difference in PAX7 expression between uninjected and injected sides. Mean number of PAX7+ cells is 42.8 on uninjected and 28.6 on CDH11MO-injected side, *p* = 0.02, *n* = 18. Mean number of PAX7+ cells is 47.77 on uninjected and 57.23 on CDH11MO + CDH11-GFP-injected side, *p* = 0.15, *n* = 13. Mean number of PAX7+ cells is 35.06 on uninjected and 39.94 on CDH11MO + p53MO-injected side, *p* = 0.48, *n* = 16. **(L)** Graph showing difference in *Casp3 expression between uninjected and injected sides. Mean number of *Casp3+ cells is 17.90 on uninjected and 31.86 on CDH11MO-injected side, *p* = 0.04, *n* = 14. Mean number of *Casp3 + cells is 40.20 on uninjected and 36.80 on CDH11MO + CDH11-GFP-injected side, *p* = 0.71, *n* = 10. Mean number of *Casp3 + cells is 16.50 on uninjected and 14.17 on CDH11MO + p53MO-injected side, *p* = 0.68, *n* = 12. All graphs show mean (indicated on graph) and median (line within graph). Phenotypes were rescued by co-injection with full length CDH11 as well as by blocking p53 translation suggesting that the NC phenotype is due to cell death after loss of CDH11. Scale bars for (E,F) are as marked in (E) and (G-J) are marked in (G).

Previous studies have identified both SNAI2 and SOX9 as factors that prevent apoptosis in NC cells in *Xenopus* and chick embryos, respectively (Tribulo et al., 2004; Cheung et al., 2005). Further, increased p53 expression has been linked to reduction in SNAI2 protein expression and increased craniofacial defects in chick and mouse (Rinon et al., 2011). Due to the reduction in the expression of the NC specifier proteins and increased in p53 and *Casp3 expression after CDH11 knockdown, we hypothesized that blocking the p53-mediated apoptotic pathway would rescue the phenotype caused by reduction of CDH11. To confirm that the NC phenotype was specific to changes in CDH11 expression, we first compared embryos injected with CDH11MO alone to those co-injected with CDH11MO and full length CDH11-GFP rescue construct and performed IHC for PAX7 expression to examine NC development. As expected, whereas loss of CDH11 reduced PAX7-expressing cells at stages after HH8 significantly (Figures 5A,K, *n* = 18, *p* = 0.02), PAX7 expression was rescued by co-injection of CDH11-GFP (Figures 5B,E,F,K, *n* = 13, *p* = 0.15). We next co-injected CDH11MO with a p53 translation-blocking morpholino (p53MO) to rescue the loss of PAX7. Injection of p53MO alone had little effect on PAX7 expression (Figure 5C), but interestingly, co-injection of CDH11MO with p53MO was able to partially rescue the PAX7-positive cell population (Figures 5D,G,H,K, *n* = 16, *p* = 0.48). To confirm that the renewed PAX7 expression was caused by a reduction in CDH11-p53-mediated cell death, we verified the rescued phenotype by analyzing *Casp3 expression in CDH11MO and p53MO co-injected embryos (Figures 5I,J,L, *n* = 14, *p* = 0.04), injection of p53MO alone did not significantly change levels of *Casp3 in embryos (Figure 5L, *n* = 10, *p* = 0.71). However, blocking p53 was able to partially rescue the increased *Casp3 expression (Figures 5I,J,L, *n* = 12, *p* = 0.68). These data demonstrate that loss of CDH11 reduces NC cells due to increased p53-mediated cell death, and that blocking p53-mediated apoptosis can rescue the loss of NC cells.

### CDH11 Is Required for Normal NC Cell EMT, Morphology, and Migration

**Figure 6:**
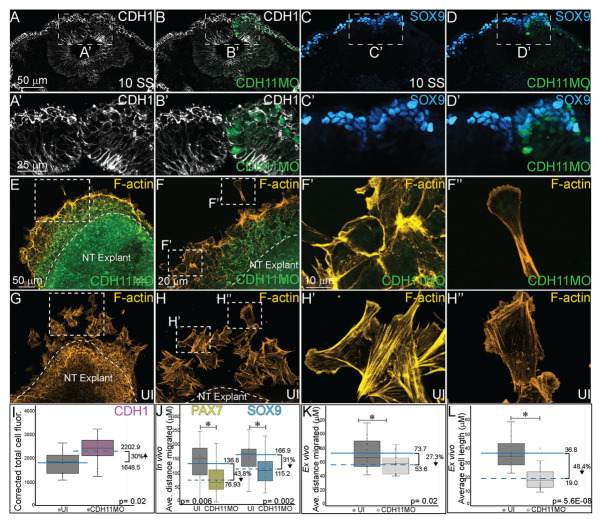
CDH11 knockdown affects cell morphology and NC cell migration. To determine if the NC determination and cell death phenotype affects cell morphology *in vivo* **(A–D’)** embryos were injected unilaterally with CDH11MO and electroporated at HH4, and IHC was performed for **(A–B’)** CDH1 to mark epithelial cells and **(C–D’)** SOX9 to mark definitive NC cells. Dashed boxes in (A-D) indicate location of zoom in from **(A’–D’)**.To determine if the CDH11 knockdown phenotype affects cell morphology and migration *ex vivo* **(E–H”)** neural tube explants were dissected from HH8 embryos, cultured on fibronectin coated slides, and stained for filamentous actin (F-actin). **(A)** IHC for CDH1 in transverse section from 10 SS embryo with **(B)** overlay with CDH11MO (green). **(A’)** Zoom in of dashed box from **(A)** showing increased CDH1 expression in dorsal neural tube in CDH11MO-injected versus uninjected side. **(B’)** Overlay with CDH11MO. **(C)** IHC for SOX9 in transverse section from the same 10 SS embryo from **(A)** with **(D)** overlay with CDH11MO (green) demonstrating reduced migration on CDH11-injected side. **(C’,D’)** Zoom in of dashed box from **(C,D)**. **(E-F”)** Staining for F-actin in explant from embryo unilaterally injected with CDH11MO and electroporated at HH4, at **(E)** 20X and **(F)** 40X magnification. **(F’)** Zoom in of single follower cell from CDH11MO-injected explant. **(F”)** Zoom in of single leading cell from CDH11MO-injected explant. Both cells are significantly closer to epithelial explant and smaller than uninjected cells. **(G–H”)** Staining for F-actin in explant from uninjected side at **(G)** 20X and **(H)** 40X magnification. **(H’)** Zoom in of grouped follower cells from uninjected explant. **(H”)** Zoom in of single leading cell from uninjected explant. **(I)** Graph showing *in vivo* difference in CDH1 between uninjected and CDH11MO-injected sides. Corrected mean total cell fluorescence of CDH1 in the dorsal neural tube is 1645.5 on uninjected and 2202.9 on CDH11MO-injected side, *p* = 0.02, *n* = 11. **(J)** Graph showing difference in migration *in vivo* from midline of PAX7 and SOX9-positive cells between uninjected and CDH11MO-injected sides. Average distance migrated away from midline by PAX7+ cells is 136.8 μm on uninjected and 76.93 μm on CDH11MO-injected side, *p* = 0.006, *n* = 11 cells. Average distance migrated away from midline by SOX9+ cells is 166.87 μm on uninjected and 115.22 μm on CDH11MO-injected side, *p* = 0.002, *n* = 19. **(K)** Graph showing average distance migrated *ex vivo* by cells from explant is 73.7 μm from uninjected explant and 53.6 μm from CDH11MO-injected explant, *p* = 0.02, *n* = 17 cells. **(L)** Graph showing average cell length is 36.8 μm in uninjected explants and 19.0 μm in CDH11MO-injected explants, *p* = 5.6E-08, *n* = 23 cells. Overall, loss of CDH11 significantly reduces NC cell population, affects their morphology, and reduces cell migration as a result. All graphs show mean (indicated on graph) and median (line within graph). Scale bars for (A-D) are as marked in (A), (A’,B’) are marked in (A’), (E,G) are marked in (E), (F,H) are marked in (F), and (F’–H”) are marked in (F’).

Finally, since cell adhesion molecules such as cadherins play a critical role in morphological changes during NC EMT and migration, we investigated how the NC cell features were affected by the early loss of CDH11 in the premigratory cells *in vivo*. To this end, we analyzed changes in the expression of E-cadherin/CDH1, a membrane-bound type I cadherin protein that has been linked to NC cell specification (Rogers et al., 2018) and to NC cell EMT and migration in both frog and chick embryos (Rogers et al., 2013; Huang et al., 2016). CDH11MO was unilaterally injected and electroporated at HH4, and cell morphology, CDH1 fluorescence intensity, and cell migration distance were measured in HH10 (10 SS) embryos. Loss of CDH1 enhanced CDH1 protein fluorescence in NC cells on the injected side (Figures 6A–B’, *n* = 11, *p* = 0.02), which resulted in defective migration while cells from the uninjected side had already migrated. We separately measured the distance from the midline that PAX7 and SOX9-positive cells traveled in CDH11MO-injected cells compared to uninjected sides because their expression differs in premigratory NC cell populations. CDH11MO-injected SOX9-positive cells migrated 31% less than the uninjected side (Figures 6C–D’,J, *n* = 17, *p* = 0.026) and PAX7-positive cells migrated 43.8% less than the uninjected side (Figure 6J, *n* = 19, *p* = 0.029). Our results suggest that CDH11 is necessary for NC specification, survival, and EMT. As a result of the early phenotype, cell morphology and migration remain affected at migratory stages.

We also performed NC explant assays to better assess the morphology and migratory ability of the cells lacking CDH11 *ex vivo* to determine if the NC migration defects in embryos are due to intrinsic or extrinsic properties. Embryos were electroporated with CDH11MO at HH4 and neural tube explants were dissected from the embryos at HH8 (3 SS–6 SS), cultured for 8 h, and then stained for filamentous actin (F-actin). Explants were repeated in triplicate and both leading edge and follower cells were measured (distance and size) individually. Cells lacking CDH11 migrated away from the explant 27.3% less than uninjected cells (Figures 6E–F”,K, *n* = 17, *p* = 0.02). The average migration distance from the epithelial explant by CDH11MO-injected cells was 53.6 μm while uninjected cells demonstrated a normal migratory ability, formed both lamellipodia and filopodia, and migrated approximately 73.7 μm from the explant (Figures 6G–H”,K). Very few cells were physically able to detach from the collective group in CDH11MO-injected explants, and most remained strongly adherent to the epithelial explant (compare Figures 6E,F’–G,H’). In addition to exhibiting migration defects, cells lacking CDH11 were significantly smaller more rounded and lacked filopodia similar to *Xenopus* NC cells lacking CDH11 (Kashef et al., 2009; compare Figures 6F’,F”-H’,H”, *n* = 23 cells, *p* = 5.6E-08).

## Discussion

**Figure 7:**
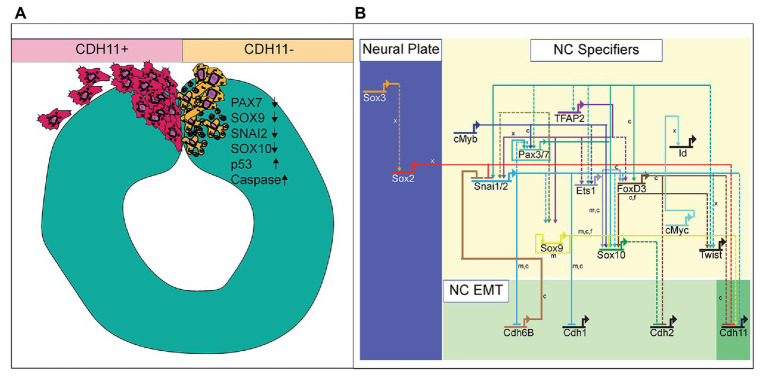
Summary diagram of CDH11-knockdown phenotype. **(A)** Image depicting NC cell development in normal NC cells (CDH11+) and cells lacking CDH11 (CDH11-). Normal cells undergo EMT, exit the neural tube, migrate collectively, and then progressively mesenchymalize as development proceeds. Normal lamellipodial and filopodial projections form as the cells navigate through the extracellular matrix. In the absence of CDH11, NC cells are induced, but undergo p53-mediated apoptosis due to either (1) inability to complete EMT/migration or (2) altered intracellular signaling in the absence of CDH11. PAX7, SOX9, SNAI2, and SOX10 positive cells are all significantly reduced in the absence of CDH11 while p53 and *Casp3 are upregulated. **(B)** Simplified NC GRN identifying NC specifiers and multiple putative inputs into the CDH11 upstream regulatory region as identified by ATAC-seq performed on NC cells (Williams et al., 2019). Little is known about the downstream targets and effectors of most cadherin proteins in the NC GRN with the exception of CDH6B (Schiffmacher et al., 2016) and CDH11 in migratory NC cells (Kashef et al., 2009; Koehler et al., 2013), and therefore identifying their targets in premigratory NC cells is essential. Direct binding relationships are indicated with solid lines while putative regulatory relationships are indicated by dashed lines. Letters indicate species in which experiments were performed: *x* = *Xenopus*, *c* = chicken, *m* = mouse. GRN information sourced from Rogers and Nie (2018).

Defining the specific roles of cell adhesion proteins in early development is necessary as abnormal expression of these proteins is linked to cellular anomalies and congenital defects. With dynamic expression profiles, differential downstream signaling; and implications in cell survival, specification, and migration, cadherin proteins are involved in multiple aspects of embryonic development. Here, we examined the localization and function of CDH11 protein in early avian NC cells. CDH11 is expressed in the neural tube prior to NC cell formation, is turned on in the NC cells at the premigratory stages, and is maintained in migratory NC cells. Loss of CDH11 reduced the number of specified cells (SNAI2, SOX9, SOX10-positive) that normally proceed to cell migration and reduced proteins that are crucial for NC cell survival (SNAI2, SOX9). Furthermore, loss of CDH11 increased levels of CDH1 in premigratory NC cells, which failed to migrate normally. In contrast to previous studies, loss of CDH11 had little effect on cell proliferation but increased p53 expression and p53-mediated cell death in the neural tube, which was either a result of losing SNAI2 and SOX9 or caused the reduction in those proteins. Inhibition of p53 in cells deficient for CDH11 partially rescued the loss of NC cells and the increase in *Casp3 (Figure 7A). Previous studies in frog embryos (Borchers et al., 2001; Kashef et al., 2009; Langhe et al., 2016) have reported that CDH11-deficient cells exhibit migratory and morphological defects. Our results shed light on the CDH11-mediated events in NC prior to migration and allows us to posit that at least some of the migratory defects may be secondarily caused by defects in cell specification and cell survival.

### Critical Timing of CDH11 Function

NC cells are a dynamic population of cells controlled by multiple levels of secreted morphogens, transcription factors, epigenetic modifiers, and cell-cell adhesion molecules (Martik and Bronner, 2017). To understand the potential role of CDH11 in early development, we characterized the spatiotemporal expression and localization of the protein *in vivo* in stages earlier than those previously identified (Chalpe et al., 2010). Our data indicate that CDH11 protein is expressed in the neuroepithelium during neurulation and is specifically upregulated above its neuroepithelial levels in the *bonafide* NC cells (SOX9+, SNAI2+) prior to EMT (Figure 1). The bulk of previous work studying CDH11 function in NC cells focused on its necessity for normal NC cell migration including its post-translational processing (McCusker et al., 2009; Abbruzzese et al., 2016), the formation of focal adhesions, protrusive activity, and extracellular matrix dynamics (Kashef et al., 2009; Langhe et al., 2016; Row et al., 2016). Here, we focused on understanding the pre-migratory role of CDH11 in NC cell development. CDH11 is expressed at very low levels in the neural plate border cells during NC induction, and our results suggest that it is not necessary for NC induction (3 SS and earlier), but rather it is necessary for NC cell specification and survival (5 SS and later; Figure 2). In support of our results, previous studies in *Xenopus* embryos showed that both exogenous CDH11 and dominant negative CDH11 expression reduced the population of undifferentiated NC cells and inhibited NC cell migration without affecting neural plate specification (Borchers et al., 2001). Interestingly, the same study reported that NC cells gained neural cell identities in the presence of dominant negative CDH11, in line with our finding that CDH11 may be necessary for specification during premigratory stages. We believe that our results fill a gap in the previously reported phenotype. Loss of CDH11 reduces the definitive NC cell populations (Figures 2, 3) because without CDH11, these cells undergo p53-mediated cell death (Figures 4, 5). The timing of cell death occurs after induction but prior to NC cell EMT and migration. Our data suggest one of two possibilities; intracellular signaling downstream of CDH11 is necessary for the maintenance and survival of the premigratory NC cells, or that the cells require the presence of CDH11 for normal cell-cell adhesion-related EMT preparatory steps prior to migration, and without it they die. Future experiments are designed to test those hypotheses. Although CDH11 and other cadherins currently reside at the end of the NC GRN and are thought to function solely as regulators of NC EMT and migration downstream of NC specifiers, recent work that characterized all open enhancers in premigratory NC cells indeed identified putative binding sites in the CDH11 enhancer for SNAI2 and SOX9 (Figure 7B; Williams et al., 2019). Their enhancer analyses suggest that SNAI2 and SOX9 may drive CDH11 expression prior to EMT while FOXD3 and SOX2 may repress it. Future work will determine which of these factors functions upstream of CDH11 in the NC cells and whether the effects of CDH11 knockdown on cell specification and survival are direct (SOX9/SNAI2➔CDH11➔cell specification/survival) or indirect through changes in NC specifiers (CDH11➔SOX9/SNAI2➔cell specification/survival).

### CDH11 and p53 Mediated Apoptosis

Our initial investigation into the role of CDH11 during early embryogenesis stemmed from its expression in developing NC cells and the varied accounts of its function in cancer cells. Previous studies in embryos demonstrated that CDH11 is necessary for NC migration (Vallin et al., 1998; Kashef et al., 2009; Abbruzzese et al., 2016; Langhe et al., 2016), but very few studies dissected its function in formation or survival *in vivo* (Borchers et al., 2001). Additionally, the role of CDH11 in cancer cells is variable. In murine retinoblastoma, CDH11 functions as a tumor suppressor, and overexpression of the gene in mouse models resulted in increased cell death (Marchong et al., 2010). This study relates to our data demonstrating that changes in CDH11 induces cell death or reduces NC cell survival; however, the mechanisms driving cell death downstream of gain and loss of CDH11 are likely unique in each situation. Both SLUG/SNAI2 (Tribulo et al., 2004) and SOX9 (Cheung et al., 2005) have demonstrated anti-apoptotic activity in NC cells, and their presence in the neural folds is correlated with reduced cell death. The previous studies paired with our data showing that loss of CDH11 reduces SOX9 and SNAI2 expression while concurrently increasing p53 expression (Figures 3, 5), suggests a link between CDH11 and the p53-mediated apoptotic pathway.

However, rather than driving cell death indirectly, it is possibly based on its requirement for CDH11 in NC migration. The loss of CDH11, whether through changes in cell-cell adhesion or intracellular signaling, drives cell death. Loss of CDH11 in the premigratory cells creates a defect in the mechanisms driving NC delamination and EMT. Western blot analysis using protein lysates from progressives stages of chicken embryos confirmed that chicken CDH11 undergoes processing and cleavage similar to *Xenopus* embryos (Supplementary Figure S1A), likely creating an extracellular fragment that would interact with the full length CDH11 to drive NC cell migration (McCusker et al., 2009). In our study, the NC cells have activated NC specifier proteins (SOX9, SNAI2, SOX10) and attempt to migrate, but cannot due to abnormal levels of CDH1 that prevent proper mesenchymalization (Figure 6; Rogers et al., 2013). Cells lacking CDH11 have increased F-actin cabling (Figure 6), which has previously been linked to the execution phase of cell death and is required for the formation of apoptotic bodies in embryonic carcinoma cells (Neradil et al., 2005). Additionally, actin-induced activation of the Ras signaling pathway has been previously linked to apoptosis (Gourlay and Ayscough, 2006). In contrast, overexpression of CDH11 may function to activate cell death *via* a Wnt-or Rho-dependent mechanism as previously described (Li et al., 2012; Row et al., 2016), and future experiments are designed to identify CDH11-specific apoptotic mechanisms.

### Implications and Considerations for Disease Studies

Contrasting conclusions about the role of CDH11 in the disease state may be clarified if its function is assessed in the context of epithelial vs. mesenchymal cells. Loss of CDH11 prior to NC cell migration prevents the cells from leaving the neural tube efficiently, thereby activating the p53-mediated apoptotic pathway. The lack of specific expression in NC progenitors and generalized neural tube expression until just prior to the stage of migration suggests that CDH11 may play another role in the developing neuroepithelium. These data support a dual role for CDH11 during development. The upregulated expression of CDH11 in premigratory NC cells at 5 SS coincides with the wild type expression of SNAI2, SOX9, and SOX10, three proteins that are necessary for NC cell migration. It also coincides with the stage at which N-cadherin is reduced in the dorsal neural tube (Rogers et al., 2018). We believe that the functional type II cadherin complex is required for the completion of EMT and that loss of CDH11 leads to an increased tension on the cytoskeletal elements of the NC cells as evidenced by the increased CDH1 expression and F-actin localization causing activation of the p53-mediated apoptotic pathway. This hypothesis is supported by previous work demonstrating tightly controlled cadherin proteins in the NC EMT process (Coles et al., 2007; Rogers et al., 2013; Schiffmacher et al., 2014, 2016; Scarpa et al., 2015). In essence, the cells are programmed to migrate, but because they are unable, they are directed to die. As altering CDH11 affects cell survival, CDH11 may be functioning as it does in cancer cells, as pro-apoptotic stemness modulator that functions *via* the WNT and Rho pathways. Future experiments will focus on understanding the specific mechanisms downstream of CDH11 that regulate the NC cell population.

### Possible Mechanisms Downstream of CDH11

In *Xenopus*, CDH11 controls filopodia and lamellipodia formation by binding to the guanine nucleotide exchange factor (GEF) proteins. Overexpression of cdc42, Rac1, and RhoA in frog embryos lacking CDH11, rescues cranial NC cell migration (Kashef et al., 2009), and the GEF proteins specifically regulate NC cell protrusion and migration likely mediated by Rac1 (Kratzer et al., 2020). Therefore, loss of CDH11 may cause alterations in intracellular signaling and cytoskeletal rearrangements specifically linked to altered Rac/Rho signaling downstream of CDH11.

CDH11 also interacts with the proteoglycan, Syndecan-4, which maintains cell-substrate adhesion during cell migration. Loss of Syndecan-4 increased Rac activity, and inhibition of Rac1 by Syndecan-4 regulated the migration of NC cells (Matthews et al., 2008; Langhe et al., 2016). However, the non-canonical Wnt Planer Cell Polarity (PCP) Pathway promotes RhoA activity, which is necessary for NC cell migration, and inhibition of RhoA increased in Rac activation which can induce cell death (Matthews et al., 2008). It is possible that if the NC phenotype caused by loss of CDH11 is not directly related to cell-cell adhesion specific migration defects, rather, CDH11 may be a novel regulator that functions between Rac1 and RhoA, and loss of CDH11 may activate Rac1, preventing the cells from migrating and inducing apoptosis. Future studies will continue to investigate the mechanisms that cause a reduction in the NC cell population in CDH11-deficient cells, but it is clear from our studies and others that CDH11 plays a complex and important role in NC cell formation and survival prior to its role in migration.

Loss of CDH11 causes p53-mediated cell death and reduces the number of *bonafide* NC cells in addition to causing morphological and migratory defects both *in vivo* and *ex vivo*. Our analyses add new information to previous discoveries, demonstrating that CDH11 is not solely required for migration, but plays an important role prior to NC cell emigration from the neural tube.

## Materials and Methods

### Chicken Embryos

Fertilized chicken eggs were obtained from local sources (Sunstate Ranch, CA and the UC Davis Hopkins Avian Facility) and incubated at 37°C to the desired stages according to the criteria of Hamburger and Hamilton (HH). Use and experiments on embryos was approved by the California State University Northridge IACUC protocol: 1516-012a, c and the UC Davis IACUC protocol #21448.

### Microinjection and Electroporation

Translation blocking antisense fluorescein or biotin-labeled morpholinos to CDH11 (CDH11MO; 5'-TATTTTGTAGGCACAGGAGTATCCA-3'), p53 (5'-CAATGGTTCCATCTCCTCCGCCATG-3') and a non-specific control morpholino (ContMO; 5'-CCTCTTACCTCAGTTACAATTTATA-3') were microinjected into the right side of a Hamburger-Hamilton stage 4–5 chicken embryo and platinum electrodes were placed vertically across the embryos and electroporated with five pulses of 6.3–6.8 V in 50 ms at 100 ms intervals. Injections of the morpholinos (0.5 mM^-1^ mM) were paired with 0.5–1.5 mg/ml of carrier plasmid DNA (Voiculescu et al., 2008) to enhance cell uptake of treatment. Injections were performed by air pressure using a glass micropipette targeted to the presumptive neural plate and neural plate border region. DNA plasmids pCAGGS-CDH11-IRES-GFP^1^, Sirius-H2B-C-10 (injected as marker for CDH11MO) were a gift from Michael Davidson to Addgene (Addgene plasmid # 55226^2^; RRID:Addgene_55226) and were introduced in a similar manner to morpholinos described above. HH stage 4–5 electroporations were conducted on whole chick embryo explants placed ventral side up on filter paper rings.

### Immunohistochemistry

Immunohistochemistry (IHC) was performed as described previously (Strobl-Mazzulla and Bronner, 2012; Rogers et al., 2013). Briefly, for IHC, chicken embryos were fixed on filter paper in 4% paraformaldehyde (PFA) in phosphate buffer for 15–25 min at room temperature. After fixation, embryos were washed in 1X TBS (500 mM Tris-HCl, pH 7.4, 1.5 M NaCl, and 10 mM CaCl_2_) containing 0.1% Triton X-100 (TBST+ Ca2^+^). Short fixation times and TBST+ Ca2^+^ were used to enhance the IHC for cadherin proteins specifically. IHC using longer fixation or PBS + Triton without Ca2^+^ resulted in much lower antigen signal. For blocking, embryos were incubated in TBST+ Ca2^+^ and 10% donkey serum for 1 h at room temperature. Primary antibodies were diluted in blocking solution and incubated with embryos for 3 h at room temperature or for 24–48 h at 4°C. After incubation with primary antibodies, whole embryos were washed in TBST + Ca2+, incubated with AlexaFluor secondary antibodies diluted in blocking buffer (1,500) for 3 h at room temperature or 12–24 h at 4°C. They were then washed in TBST+ Ca2^+^, and post-fixed in 4% PFA for 30 min^−1^ h at room temperature. Antibodies used in the study (Table 1): Rabbit α-Cadherin-11 (Cell Signaling Technologies, #4442), Mouse α-Cadherin-11 (Invitrogen, #5B2H5), Mouse α-E-cadherin (BD Transduction Laboratories, 61081), Rabbit α-Active Caspase-3 (R and D Systems, #AF835), Mouse α-PAX7 (DSHB), Rabbit α-SOX9 (EMD Millipore, #ab5535), Rabbit α-SOX2 (Abcam, #ab97959), Mouse α-SOX10 (Proteintech, #66786-1-Ig), Rabbit α-SLUG/SNAI2 (Cell Signaling Technology, #9585S), Mouse α-p53 (Millipore, CBL404), and Rabbit α-Phospho histone H3 (R7D Systems, #ab5176). After IHC all embryos were imaged in both whole mount and transverse section (after cryosectioning) using a Zeiss Imager M2 with Apotome capability and Zen optical processing software.

**Table 1:** Antibodies used in study.

Antibody	Dilution	SOURCE	IDENTIFIER
Mouse monoclonal anti-CDH11 (CDH11/Cadherin-OB) IgG1	1:250 IHC/1:10,000 WB	Invitrogen	5B2H5
Rabbit polyclonal anti-CDH11 (CDH11/OB Cadherin) IgG	1:200 IHC/1:10,000 WB	Cell Signaling Technologies	4442
Mouse anti-Pax7 IgG1	1:5	DSHB	PAX7
Mouse anti-HNK1 IgM	1:5	DSHB	3H5
Mouse anti-CDH1 IgG2a	1:500	BD Biosciences	610181
Rabbit anti-SOX2 IgG	1:250	Abcam	ab97959
Rabbit anti-SOX9	1:500	EMD Millipore	ab5535
Rabbit anti-SLUG (SNAI2)	1:250	Cell Signaling Technology	9585S
Mouse anti-SOX10 IgG2a	1:500	Proteintech	66786-1-Ig
Rabbit anti-Phospho histone H3	1:500	Abcam	ab5176
Mouse anti-p53	1:250	Millipore	CBL404
Rabbit anti-Active Caspase-3	1:500	R and D Systems	AF835

### Western Blot

Embryo lysate was isolated from 10 to 20 manually dissected chicken embryos from stages HH4–6, HH8–10, and HH11–12 or tailbud stage axolotl embryos for Western blot analysis. Lysate was isolated using lysis buffer: 50 mM Tris-HCL pH 7.4 with 150 mM NaCl plus 1.0% NP-40 and EDTA-free protease inhibitor (Roche cOmplete, #11697498001). SDS page was run on precast 8–12% bis-tris gel (Invitrogen, #NP0321BOX) for 3 h at 60 V; gel was transferred to nitrocellulose using the Invitrogen iBlot2 Dry Blotting System. Nitrocellulose membranes were washed in TBST+ Ca2^+^, blocked and incubated with primary antibody in TBST+ Ca2^+^ with 5.0% milk or 5.0% BSA, were then incubated in (5%) milk protein in TBST+ Ca2^+^ with secondary antibodies, and visualized using Prometheus ProSignal Femto ECL Reagent (#: 20-302B) and exposed to Prometheus ProSignal ECL Blotting Film, 5 × 7 in. (#: 30-507 L).

### Imaging and Fluorescence Quantification

Fluorescence images were taken using Zeiss ImagerM2 with Apotome.2 and Zen software (Karl Zeiss). Fluorescence was quantified using NIH ImageJ by averaging the relative intensity of 1–6 images per embryo. Specifically, when multiple section images were available, intensity would be measured individually and then averaged over one individual. N’s represent unique individuals. Background was subtracted uniformly across the images using the background subtraction function in NIH ImageJ with a rolling-ball radius of 50.00 pixels before quantitation (Hutchins and Szaro, 2013). For *Casp3, fluorescence was compared between half neural tubes while all other analyses compared fluorescence in the dorsal half of the neural tubes on the injected and uninjected sides. Half embryos injected with CDH11MO or ContMO were compared to the uninjected or control side.

### *Ex vivo* Neural Tube Explants

For explant assays, embryos were electroporated with 0.75 mM CDH11MO plus Sirius carrier DNA on the right side of the embryo at HH4. Embryos were cultured until HH8 as described previously (Sauka-Spengler and Barembaum, 2008). At HH8, the neural tubes were dissected out of the embryo in Ringer’s solution and subsequently placed in 8-well chamber slides (Millicell EZ SLIDE 8-well glass, sterile, # PEZGS0816) that were coated with 100 μg/ml fibronectin. The explants were cultured in DMEM with 10% FBS, 2 mM l-glutamine, and 100 units of penicillin with 0.1 mg/ml streptomycin at 37°C with 0.5% CO_2_ for 8 h. After incubation, explants were fixed using 4% PFA, washed in TBST + Ca2+, and incubated with Phalloidin stain. Cytoskeletal stain was: Invitrogen Molecular Probes Alexa Fluor 568 Phalloidin (#A12380).

### TUNEL Assay

For TUNEL assay, the Click-iT Plus TUNEL Alexa Fluor 488 and 647 were used. Protocol was as the manufacturer suggests. Briefly, embryos were fixed for 15 min in 4% PFA in phosphate buffer, washed in TBST + Ca2+, washed with deionized H2O, incubated with TdT reaction mix at 37°C, washed and incubated with Click-iT reaction for 30–60 min at room temperature.

### Cell Counts and Statistical Analysis

All experiments were repeated three to four times. Cell counts and fluorescence intensity represented in box plots were either performed manually in Adobe Photoshop or were performed using NIH ImageJ. Cell counts were averaged from one to three sections per embryo. Mean, median, and standard deviation were calculated using Microsoft Excel across all biological replicates. The value of *p* was calculated in Microsoft excel using a Student’s *t*-Test with a 2-tailed distribution with unequal variance between samples for stringency. *p*-values under 0.05 are considered statistically significant. All cell counts are availables in Supplementary Tables 1–7.

## Data Availability Statement

The original contributions presented in the study are included in the article/Supplementary Material, further inquiries can be directed to the corresponding author.

## Ethics Statement

The animal study was reviewed and approved by California State University IACUC UC Davis IACUC.

## Author Contributions

Conceptualization: CR and AC-M. Data Curation: AC-M (functional experiments and imaging), SM (characterization, functional experiments, and imaging), CR (functional experiments, data analysis, and imaging), and CE (functional experiments and imaging). Formal Analysis (cell counts, statistics): AC-M, SM, and CR. Funding Acquisition: CR (funds were provided either by NIH R15 HD092170-01 from the NICHD, CSUN startup funding, and UC Davis startup funding). Methodology: AC-M, SM, and CR. Supervision: CR. Writing: CR (wrote manuscript and performed editing), SM (wrote manuscript and performed editing), and AC-M (performed editing). All authors contributed to the article and approved the submitted version.

### Conflict of Interest

The authors declare that the research was conducted in the absence of any commercial or financial relationships that could be construed as a potential conflict of interest.

## Footnotes

^1^VectorBuilder.com

^2^http://n2t.net/addgene:55226
