# Supplementary material for: Cadherin-11 Is Required for Neural Crest Specification and Survival
Source: Front Physiol. 2020 Oct 30;11:563372. doi: 10.3389/fphys.2020.563372 (PMC7662130; doi:10.3389/fphys.2020.563372)
Supplement: Supplementary file 1 [file Data_Sheet_1.pdf]

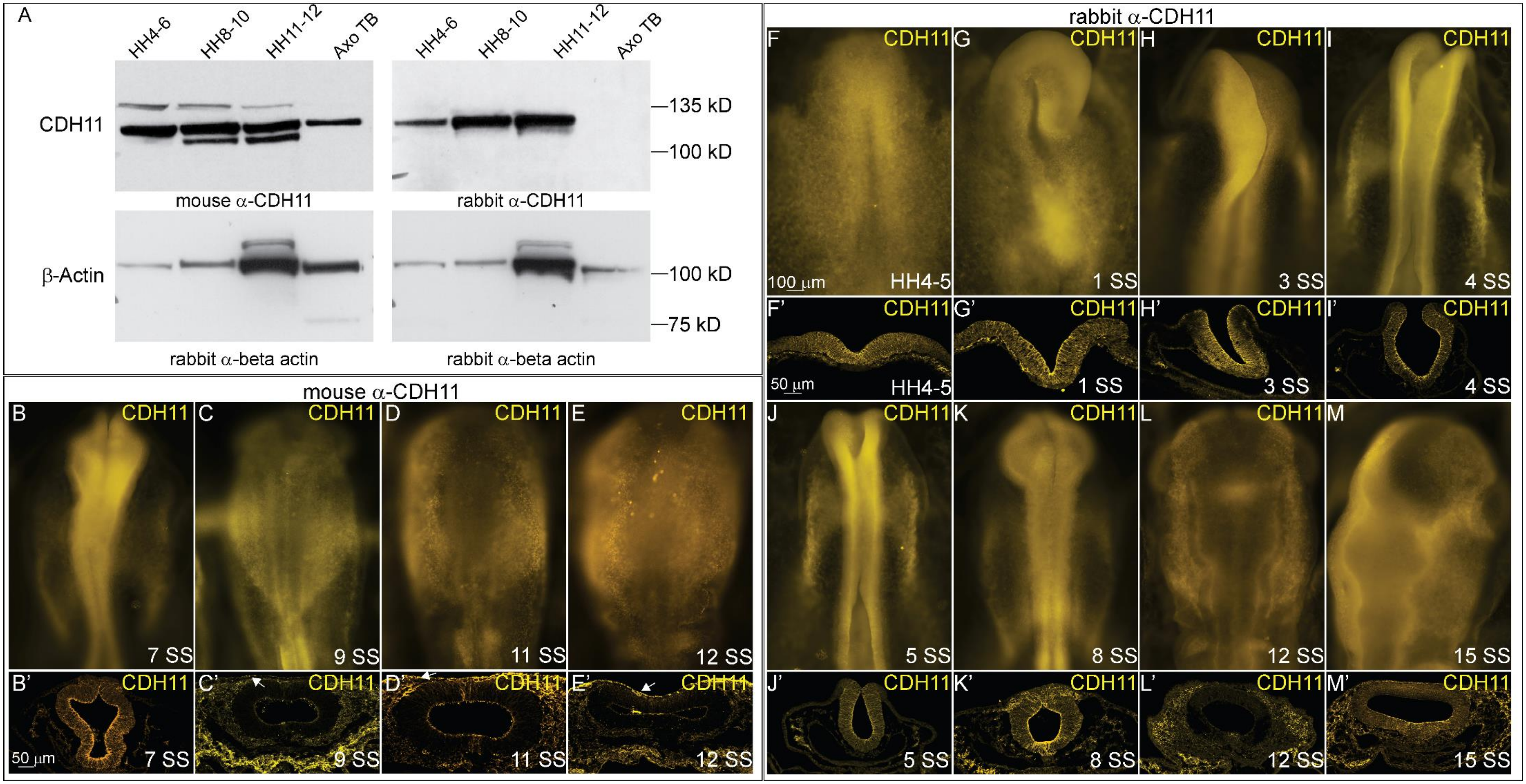

Supp. Fig. 1. CHD11 expression in whole mount and sections. To identify spatiotemporal localization in whole mount and in transverse sections, western blot analysis and IHC for CDH11 in protein lysate and uninjected embryos was performed. (A) Western blot analysis using protein lysate isolated from 10 pooled chick or axolotl embryos from stages: HH4-6, HH8-10, HH11-12, and tailbud axolotl embryos. Two antibodies were tested, mouse anti-CDH11 IgG1 or rabbit anti-CDH11 IgG. (B-I) Whole mount and (B'-I' transverse sections of (B-I) after IHC for CDH11 in (B, B') an HH4-5 embryo, (C, C') a 1 SS embryo, (D, D') a 3 SS embryo, (E, E') a 4 SS embryo, (F, F') a 5 SS embryo, (G, G') a 6 SS embryo, (H, H') a 12 SS embryo and (I, I') a 15 SS embryo. Scale bars are as marked (100 µm for whole mount and 50 µm for sections).

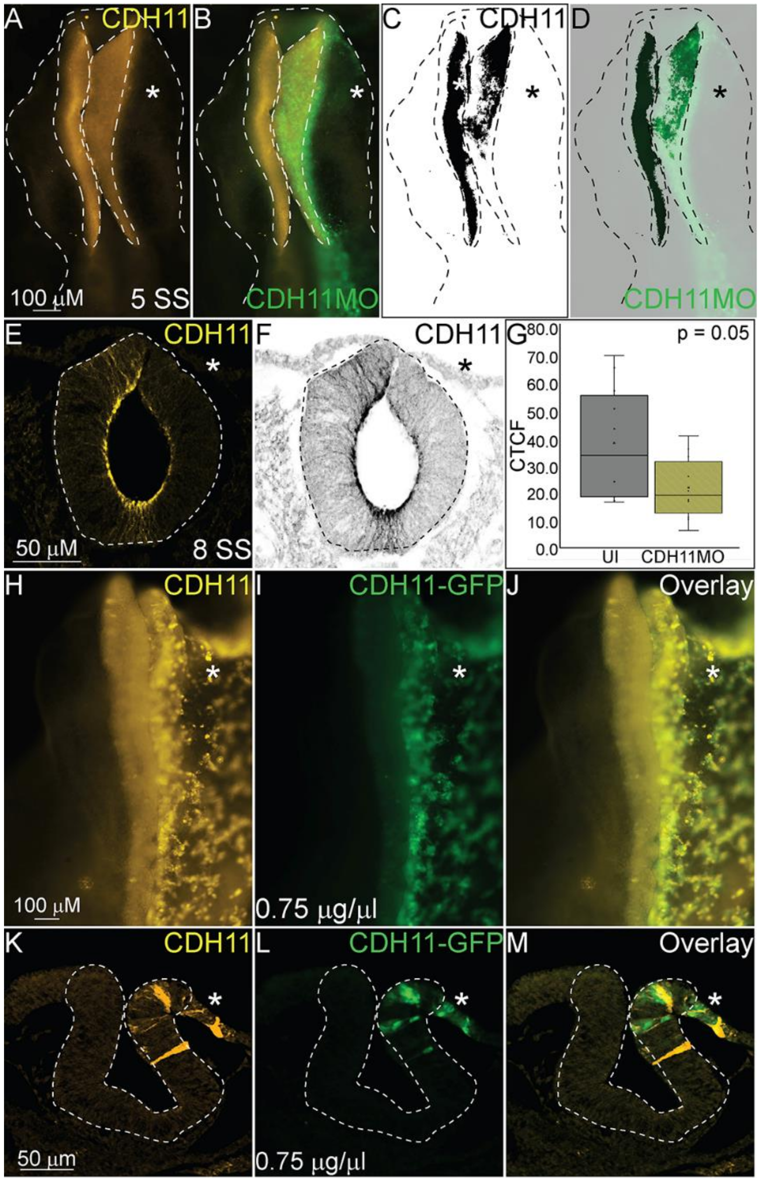

Supp. Fig. 2. Efficiency of knockdown and overexpression tools. To verify the efficiency and functionality of CDH11 translation blocking MO and CDH11-GFP full length expression vector, embryos were injected with either treatment and IHC was performed for CHD11. (A-G) IHC for CDH11 after CDH11MO electroporation at stage 4 shows that the CDH11MO efficiently inhibits the translation of CDH11 in the injected region of the neural tube. (A) Whole mount embryo after IHC for CDH11 and (B) overlay with CDH11MO (green) at 5 SS. (C) CDH11 threshold for fluorescence intensity analysis in whole mount embryos and (D) overlay with CDH11MO. Transverse section of 8 SS embryo showing IHC for CDH11 in (E) yellow and (F) grayscale. (G) Graph demonstrating CDH11 fluorescence intensity in uninjected vs. CDH11MO-injected sides of the neural tube (average fluorescence intensity of uninjected side is 39.33 and CDH11MO-injected side is 23.32, p= 0.05, n= 10). CDH11-GFP was electroporated into stage 4 embryos at 0.5 mg/ml and 0.75 mg/ml, and (H-J) IHC using for CDH11 shows that the CDH11-GFP ectopically expresses CDH11 in the neural tube. (K-M) Section of (H-J) after 0.75 mg/ml CDH11-GFP overexpression. Scale bars are as marked (100 µm for whole mount and 50 µm for sections).

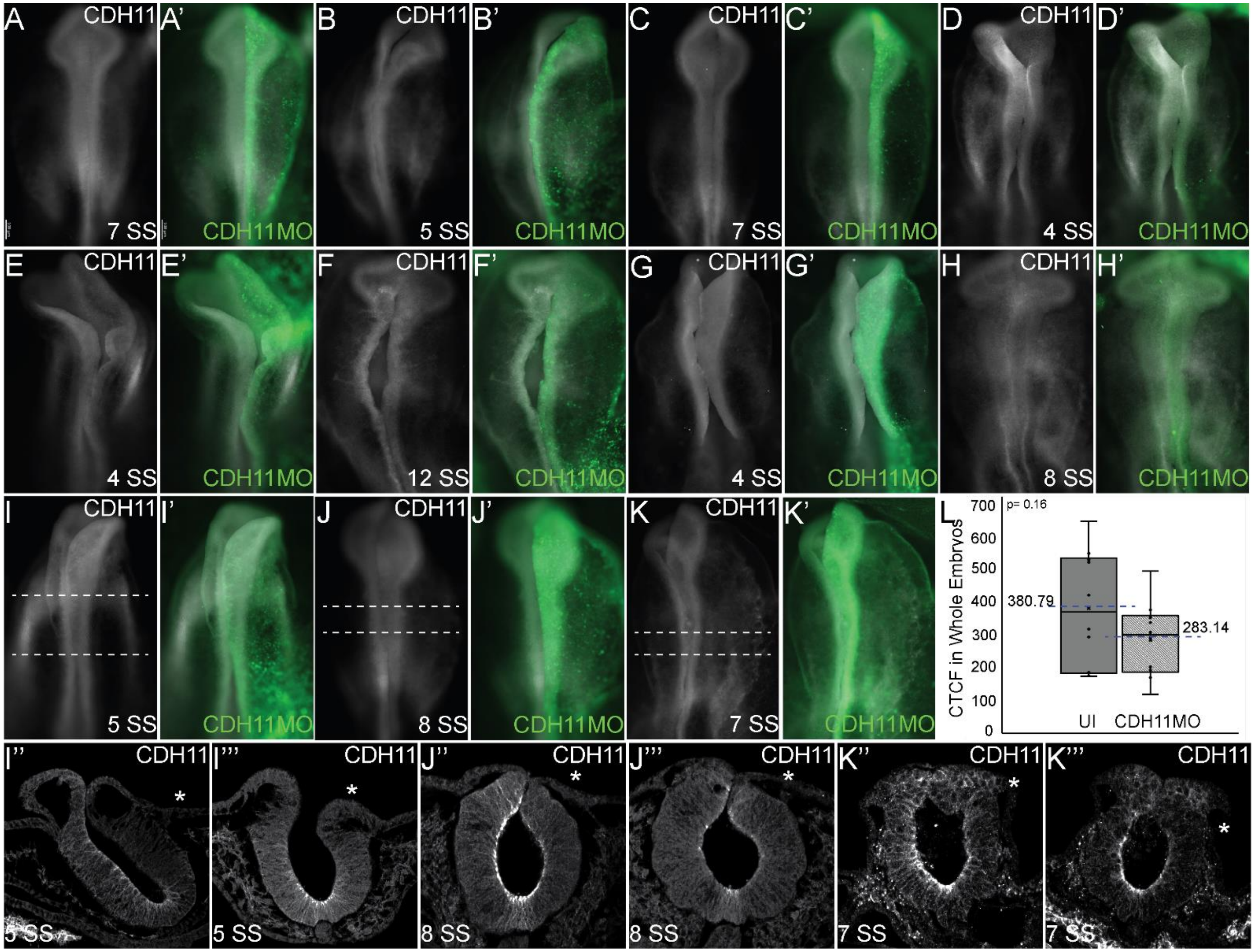

Supp. Fig. 3. Efficiency of CDH11 knockdown in whole mount and at various axial levels. To further confirm the efficiency and functionality of CDH11 translation blocking MO we measured fluorescence intensity in (A-L) multiple whole mount embryos using ImageJ, and found that due to the ectoderm-specific CDH11 knockdown and the internal (neural tube-specific) changes in expression after knockdown, CDH11 fluorescence levels were not significantly reduced when comparing the uninjected vs. CDH11MO-injected sides of the whole embryo (average fluorescence intensity of uninjected side is 380.79 and CDH11MO-injected side is 283.114, p= 0.16, n= 10) as they were in the transverse sections (Fig. S2). To verify that the knockdown remained penetrant at different axial levels, (I'-K''') transverse sections from two regions in the cranial neural tube were visualized and the reduction in CDH11 protein is more evident in this orientation.

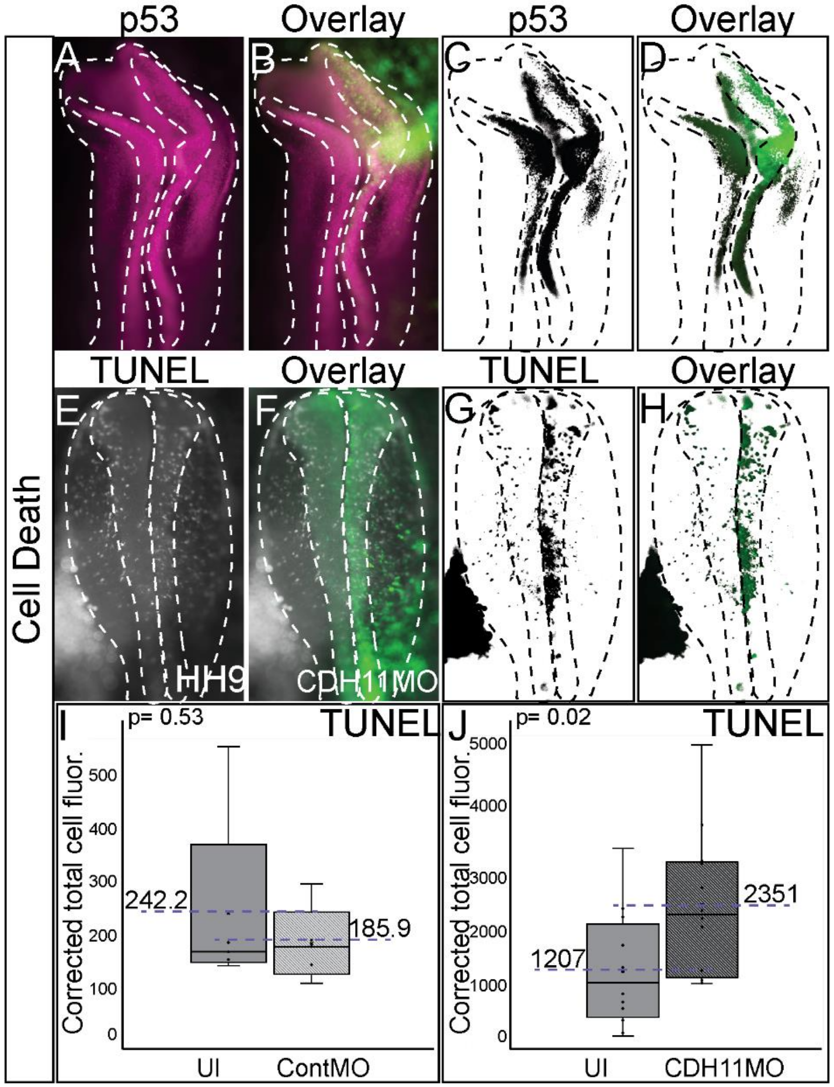

Supp. Fig. 4. CDH11 knockdown increases p53 and TUNEL expression. Embryos injected with CDH11MO at HH4 were fixed and IHC or TUNEL assay was performed in whole mount. (A) IHC for p53 and (B) overlay with CDH11MO in whole embryo. (C) p53 threshold image and (D) overlay with CDH11MO. (E) TUNEL assay in whole embryo, (F) overlay with CDH11MO (G) TUNEL threshold image and (H) overlay with CDH11MO. (I) Graph showing corrected total cell fluorescence in uninjected vs. ContMO-injected (n= 12, p= 0.53) and (J) shows TUNEL in uninjected vs. CDH11MO-injected embryos (n= 12, p= 0.02).

Supplemental Table 1. Changes in number of PAX7-expressing cells at different stages.

HH5				HH8-				HH8				HH9+				Control			
UI	Ave. # cells per 2-3 serial sections	CDH11MO	Ave. # cells per 2-3 serial sections	UI	Ave. # cells per 2-3 serial sections	CDH11MO	Ave. # cells per 2-3 serial sections	UI	Ave. # cells per 2-3 serial sections	CDH11MO	Ave. # cells per 2-3 serial sections	UI	Ave. # cells per 2-3 serial sections	CDH11MO	Ave. # cells per 2-3 serial sections	UI	Ave. # cells per 2-3 serial sections	ContMO	Ave. # cells per 2-3 serial sections
1	27	1	29	1	17	1	19	1	50	1	19	1	58	1	37		32		34
2	22	2	18	2	10	2	3	2	54	2	9	2	54	2	32		34		35
3	16	3	16	3	23	3	3	3	55	3	43	3	51	3	29		34		33
4	23	4	22	4	23	4	11	4	54	4	43	4	64	4	36		34		23
5	15	5	14	5	23	5	16	5	59	5	44	5	57	5	39		56		54
6	20	6	11	6	21	6	18	6	38	6	17	6	71	6	62		56		47
				7	21	7	13	7	88	7	43	7	37	7	21		50		49
				8	24	8	15	8	83	8	39						32		23
				9	16	9	20	9	43	9	22						14		15
				10	20	10	15	10	55	10	32						25		20
				11	20	11	40	11	31	11	10						23		25
				12	28	12	34	12	32	12	17						33		41
				13	28	13	48	13	29	13	22						36		41
				14	26	14	41	14	29	14	21						43		41
								15	54	15	32								
								16	51	16	29								
								17	64	17	36								
								18	57	18	39								
								19	15	19	51								
Mean	20.50		18.33	Mean	21.43		21.14	Mean	49.53		29.89	Mean	56.00		36.57	Mean	35.86		34.36
Median	21.00		17.00	Median	22.00		17.00	Median	54.00		32.00	Median	57.00		36.00	Median	34.00		34.50
Standard	4.51		6.41	Standard	4.85		14.09	Standard	18.16		12.60	Standard	10.68		12.74	Standard	12.00		11.84

Deviation				Deviation				Deviation				Deviation				Deviation			
Student's T-Test (2 tails, type 3)	0.52			Student's T-Test (2 tails, type 3)	0.94			Student's T-Test (2 tails, type 3)	0.0005			Student's T-Test (2 tails, type 3)	0.01			Student's T-Test (2 tails, type 3)	0.74		

Supplemental Table 2. Changes in number of PAX7, SOX9, SNAI2, and SOX10-expressing cells.

PAX7				PAX7				SOX9				SNAI2				SOX10			
UI	Ave. # cells per 2-3 serial sections	CDH11MO	Ave. # cells per 2-3 serial sections	UI	Ave. # cells per 2-3 serial sections	ContMO	Ave. # cells per 2-3 serial sections	UI	Ave. # cells per 2-3 serial sections	CDH11MO	Ave. # cells per 2-3 serial sections	UI	Ave. # cells per 2-3 serial sections	CDH11MO	Ave. # cells per 2-3 serial sections	UI	Ave. # cells per 2-3 serial sections	CDH11MO	Ave. # cells per 2-3 serial sections
1	40.33	1	15.67	1	32.00	1	34.00	1	39.00	1	22.00	1	24.00	1	15.00	1	23.00	1	19.00
2	51.00	2	48.33	2	34.00	2	35.00	2	21.00	2	9.00	2	22.00	2	5.00	2	26.00	2	14.00
3	38.00	3	17.00	3	34.00	3	33.00	3	17.00	3	6.50	3	27.00	3	14.00	3	27.00	3	14.00
4	16.50	4	3.00	4	34.00	4	23.00	4	41.67	4	19.00	4	25.00	4	14.00	4	22.00	4	10.00
5	47.00	5	3.00	5	56.00	5	54.00	5	7.50	5	7.00	5	26.00	5	12.00	5	16.00	5	17.00
6	40.33	6	21.67	6	56.00	6	47.00	6	15.25	6	12.50	6	17.00	6	8.00	6	23.00	6	16.00
7	28.67	7	14.33	7	50.00	7	49.00	7	12.83	7	8.50	7	10.00	7	14.00	7	11.00	7	2.00
8	22.00	8	15.33	8	32.00	8	23.00	8	9.50	8	5.50	8	17.00	8	17.00	8	9.00	8	1.00
9	85.50	9	41.00	9	14.00	9	15.00	9	14.00	9	12.33	9	23.00	9	9.00	9	9.00	9	1.00
10	24.67	10	21.00	10	25.00	10	20.00	10	16.50	10	12.50	10	16.00	10	7.00	10	6.00	10	3.00
11	56.50	11	34.00	11	23.00	11	25.00	11	29.50	11	15.50	11	13.00	11	6.00	11	6.00	11	3.00
12	71.00	12	62.00	12	33.00	12	41.00					12	29.00	12	9.00	12	9.67	12	4.58
13	37.00	13	21.00	13	36.00	13	41.00					13	25.00	13	18.00	13	27.00	13	9.00
14	41.50	14	25.00	14	43.00	14	41.00					14	42.00	14	31.00	14	20.00	14	10.00
15	64.00	15	49.50									15	29.00	15	21.00	15	18.00	15	17.00
16	27.00	16	44.50									16	19.00	16	20.00	16	17.00	16	11.00
17	21.00	17	41.67													17	37.00	17	19.00
18	58.00	18	37.00													18	30.00	18	26.00
																19	50.00	19	29.00

Mean	42.78		28.61	Mean	35.86		34.36	Mean	20.34		11.85	Mean	22.75		13.75	Mean	20.35		11.87
Median	40.33		23.33	Median	34.00		34.50	Median	16.50		12.33	Median	23.50		14.00	Median	20.00		11.00
Standard	18.82		16.78	Standard	12.00		11.84	Standard Deviation	11.48		5.29	Standard Deviation	7.63		6.75	Standard Deviation	11.29		8.28
Student's	0.02			Student's	0.74			Student's T-Test (2	0.04			Student's T-Test (2	0.001			Student's T-Test (2	0.01		

Supplemental Table 3. Changes in number of Caspase-expressing apoptotic bodies and PH3-cells.

CASPASE				CASPASE				PH3				PH3			
UI	Ave. # apoptotic bodies per 2-3 serial sections	Cad11MO	Ave. # apoptotic bodies per 2-3 serial sections	UI	Ave. # apoptotic bodies per 2-3 serial sections	ContMO	Ave. # apoptotic bodies per 2-3 serial sections	UI	Ave. # cells per 2-3 serial sections	Cad11MO	Ave. # cells per 2-3 serial sections	UI	Ave. # cells per 2-3 serial sections	ContMO	Ave. # cells per 2-3 serial sections
1	2.00	1	46.00	1	13.00	1	17.00	1	1.67	1	5.00	1	5.00	1	4.00
2	7.00	2	28.00	2	4.00	2	8.00	2	4.00	2	3.75	2	15.00	2	7.00
3	37.50	3	31.00	3	2.00	3	6.00	3	5.00	3	2.50	3	3.00	3	3.00

4	18.67	4	29.67	4	4.00	4	6.00	4	8.67	4	3.67	4	7.00	4	6.00
5	14.67	5	27.33	5	5.00	5	10.00	5	3.33	5	2.00	5	3.00	5	1.00
6	4.67	6	8.00	6	5.00	6	8.00	6	1.67	6	1.33	6	1.00	6	2.00
7	2.50	7	19.50	7	5.00	7	9.00	7	0.50	7	1.50	7	6.00	7	7.00
8	30.00	8	68.50	8	10.00	8	13.00	8	2.50	8	2.50	8	7.00	8	8.00
9	10.00	9	16.00	9	10.00	9	9.00	9	0.00	9	6.00				
10	46.00	10	52.00	10	16.00	10	3.00	10	8.50	10	6.00				
11	3.00	11	15.00	11	13.00	11	10.00	11	17.00	11	17.00				
12	33.00	12	62.00	12	20.00	12	14.00	12	0.00	12	2.00				
13	20.67	13	37.00	13	9.00	13	8.00	13	2.00	13	4.00				
14	23.00	14	6.00	14	15.00	14	9.00	14	7.33	14	11.33				
								15	4.00	15	2.33				
								16	14.33	16	21.67				
								17	9.50	17	10.50				
								18	12.00	18	11.33				
								19	3.67	19	5.33				
								20	0.00	20	5.00				
Mean	18.05		31.86	Mean	9.36		9.29	Mean	5.28		6.24	Mean	5.88		4.75
Median	16.67		28.83	Median	9.50		9.00	Median	3.83		4.50	Median	5.50		5.00
Standard Deviation	14.31		19.32	Standard Deviation	5.44		3.54	Standard Deviation	4.97		5.48	Standard Deviation	4.26		2.60

Student's T-Test (2 tails, type 3)	0.042			Student's T-Test (2 tails, type 3)	0.97			Student's T-Test (2 tails, type 3)	0.57			Student's T-Test (2 tails, type 3)	0.54		

Supplemental Table 4. Changes in number of PAX7-expressing cells after rescue.

PAX7				PAX7				PAX7			
UI	Ave. # cells per 1-3 serial sections	CDH11 MO	Ave. # cells per 1-3 serial sections	UI	Ave. # cells per 2-3 serial sections	CDH11MO +CDH11	Ave. # cells per 1-3 serial sections	UI	Ave. # cells per 1-3 serial sections	CDH11MO +p53MO	Ave. # cells per 1-3 serial sections
1	40.33	1	15.67	1	37.00	1	62.00	1	54.00	1	19.00
2	51.00	2	48.33	2	41.00	2	58.00	2	61.00	2	6.00
3	38.00	3	17.00	3	53.00	3	64.00	3	33.00	3	22.00
4	16.50	4	3.00	4	46.00	4	51.00	4	38.00	4	34.00
5	47.00	5	3.00	5	45.00	5	46.00	5	21.00	5	40.00
6	40.33	6	21.67	6	39.00	6	34.00	6	26.00	6	35.00
7	28.67	7	14.33	7	80.00	7	31.00	7	47.00	7	67.00
8	22.00	8	15.33	8	50.00	8	39.00	8	43.00	8	65.00
9	85.50	9	41.00	9	38.00	9	50.00	9	7.00	9	10.00
10	24.67	10	21.00	10	49.00	10	63.00	10	4.00	10	8.00
11	56.50	11	34.00	11	63.00	11	101.00	11	41.00	11	40.00
12	71.00	12	62.00	12	39.00	12	62.00	12	48.00	12	79.00
13	37.00	13	21.00	13	41.00	13	83.00	13	31.00	13	44.00
14	41.50	14	25.00					14	40.00	14	53.00
15	64.00	15	49.50					15	39.00	15	66.00

16	27.00	16	44.50					16	28.00	16	51.00
17	21.00	17	41.67								
18	58.00	18	37.00								
Mean	42.78		28.61	Mean	47.77		57.23	Mean	35.06		39.94
Median	40.33		23.33	Median	45.00		58.00	Median	38.50		40.00
Standard Deviatio	18.82		16.78	Standard Deviatio	12.15		19.33	Standard Deviatio	15.47		22.75
Student's T-Test [illegible]	0.02			Student's T-Test	0.15			Student's T-Test [illegible]	0.48		

Supplemental Table 5. Changes in fluorescence and migration distance in vivo and cell size and migration ex vivo.

CDH1				PAX7				SOX9				Explant: Cell Size				Explant: Distance Migrated			
UI	Corrected total cell fluorescence	CDH 11MO	Corrected total cell fluorescence	UI	Distance migrated (µm)	CDH 11MO	Distance migrated (µm)	UI	Distance migrated (µm)	CDH 11MO	Distance migrated (µm)	UI	Cell length (µm)	CDH 11MO	Cell length (µm)	UI	Distance migrated (µm)	CDH 11MO	Distance migrated (µm)
1	1425.13	1	1600.39	1	144.70	1	74.95	1	189.11	1	103.62	1	33.03	1	14.72	1	86.79	1	78.82
2	1865.40	2	2761.33	2	103.02	2	50.01	2	170.39	2	65.80	2	22.75	2	31.56	2	67.63	2	85.84
3	1125.86	3	1274.54	3	159.30	3	70.19	3	121.43	3	91.39	3	36.63	3	21.34	3	78.59	3	67.02

4	1367.55	4	1488.37	4	162.43	4	72.81	4	36.33	4	32.01	4	28.30	4	23.67	4	100.18	4	59.00
5	1846.43	5	2125.36	5	86.73	5	56.88	5	181.28	5	168.75	5	57.43	5	16.05	5	66.13	5	80.04
6	1400.92	6	2258.14	6	154.02	6	119.48	6	169.58	6	133.15	6	59.09	6	11.19	6	47.44	6	48.79
7	1275.91	7	2657.57	7	189.67	7	142.44	7	152.03	7	149.30	7	30.04	7	17.78	7	48.78	7	44.94
8	2157.31	8	2438.92	8	188.96	8	39.27	8	160.64	8	136.67	8	37.75	8	12.75	8	55.61	8	41.47
9	2371.54	9	3222.17	9	247.83	9	206.01	9	253.09	9	215.33	9	25.42	9	9.22	9	87.52	9	43.68
10	2646.83	10	2648.73	10	199.09	10	106.24	10	102.43	10	62.28	10	22.90	10	11.73	10	94.68	10	56.76
11	2067.15	11	2850.50	11	142.59	11	61.82	11	124.75	11	73.99	11	52.54	11	10.83	11	86.54	11	67.78
12	1958.55	12	2772.24					12	187.73	12	111.10	12	35.18	12	12.60	12	43.16	12	59.58
13	2147.77	13	2160.19					13	185.59	13	123.45	13	23.49	13	30.26	13	63.05	13	58.67
								14	154.84	14	110.03	14	41.85	14	13.89	14	94.66	14	57.61
								15	185.65	15	142.77	15	49.38	15	17.84	15	61.42	15	41.96
								16	180.16	16	86.04	16	36.34	16	19.07	16	54.41	16	42.75
								17	317.04	17	232.10	17	34.79	17	26.30	17	116.65	17	61.65
								18	176.29	18	104.15	18	43.96	18	13.06				
								19	122.12	19	47.26	19	32.89	19	40.49				
												20	28.61	20	21.18				
												21	34.43	21	15.61				
												22	45.47	22	20.22				

												23	34.74	23	26.44				
Mean	1819.72		2327.57	Mean	161.67		90.92	Mean	166.87		115.22	Mean	36.83		19.03	Mean	73.72		58.61
Median	1865.40		2438.92	Median	159.30		72.81	Median	170.39		110.03	Median	34.79		17.78	Median	67.63		58.67
Standard Deviation	466.03		584.50	Standard Deviation	44.86		49.30	Standard Deviation	57.52		52.45	Standard Deviation	10.47		7.83	Standard Deviation	21.33		13.94
Student's T-Test (2 tails, type 3)	0.02			Student's T-Test (2 tails, type 3)	0.002			Student's T-Test (2 tails, type 3)	0.006			Student's T-Test (2 tails, type 3)	0.000			Student's T-Test (2 tails, type 3)	0.02		

Supplemental Table 6. Changes in p53 and TUNEL fluorescence intensity.

p53-Relative Fluorescence				TUNEL-Relative Fluorescence			
**UI**	**Corrected total cell fluorescence**	**Cad11MO**	**Corrected total cell fluorescence**	**UI**	**Corrected total cell fluorescence**	**CDH11MO**	**Corrected total cell fluorescence**
1	83.292	1	194.848	1	1650.97	1	3102.00
2	55.706	2	270.756	2	2284.32	2	2113.74
3	38.477	3	97.472	3	669.18	3	1965.99
4	30.98	4	47.83	4	1200.18	4	2639.81
5	31.68	5	45.70	5	91.00	5	1222.46
6	42.68	6	57.61	6	2138.25	6	5100.72
7	30.59	7	28.18	7	3324.80	7	3724.42
8	29.02	8	39.44	8	1263.35	8	1047.61
9	25.01	9	41.39	9	354.55	9	2250.51
10	75.79	10	94.10	10	136.55	10	993.16
11	52.165	11	53.672	11	563.65	11	995.37
12	39.783	12	55.91	12	811.90	12	3057.54

13	26.242	13	32.498				
14	60.514	14	163.064				
15	58.905	15	96.506				
16	79.92	16	120.04				
17	52.77	17	72.67				
Mean	47.85		88.92	Mean	1207.39		2351.11
Median	42.68		57.61	Median	1006.04		2182.12
Standard Deviation	19.07		65.96	Standard Deviation	985.83		1259.96
Student's T-Test (2 tails, type 3)	0.02			Student's T-Test (2 tails, type 3)	0.02		

Supplemental Table 7. Changes in number of Caspase-expressing apoptotic bodies after rescue.

Caspase				Caspase				Caspase			
UI	Ave. # apoptotic bodies per 1-3 serial sections	CDH11 MO	Ave. # apoptotic bodies per 1-3 serial sections	UI	Ave. # apoptotic bodies per 1-3 serial sections	p53 MO	Ave. # apoptotic bodies per 1-3 serial sections	UI	Ave. # apoptotic bodies per 1-3 serial sections	CDH11MO +p53MO	Ave. # apoptotic bodies per 1-3 serial sections
1	0.00	1	46.00	1	63.00	1	56.00	1	35.00	1	12.00
2	7.00	2	28.00	2	55.00	2	75.00	2	5.00	2	2.00
3	37.50	3	31.00	3	23.00	3	24.00	3	5.00	3	3.00
4	18.67	4	29.67	4	84.00	4	36.00	4	15.00	4	12.00
5	14.67	5	27.33	5	42.00	5	30.00	5	19.00	5	22.00
6	4.67	6	8.00	6	32.00	6	35.00	6	1.00	6	5.00
7	2.50	7	19.50	7	32.00	7	48.00	7	24.00	7	8.00
8	30.00	8	68.50	8	25.00	8	26.00	8	47.00	8	38.00
9	10.00	9	16.00	9	35.00	9	30.00	9	23.00	9	35.00
10	46.00	10	52.00	10	11.00	10	8.00	10	16.00	10	26.00
11	3.00	11	15.00					11	2.00	11	1.00
12	33.00	12	62.00					12	6.00	12	6.00

13	20.67	13	37.00								
14	23.00	14	6.00								
Mean	17.90		31.86	Mean	40.20		36.80	Mean	16.50		14.17
Median	16.67		28.83	Median	33.50		32.50	Median	15.50		10.00
Standard Deviatio	14.49		19.32	Standard Deviatio	21.61		18.74	Standard Deviatio	14.16		12.96
Student' s T-Test	0.04			Student' s T-Test	0.71			Student' s T-Test	0.68		
